# KCC2 Dysfunction Mediated by Microglial BDNF/TrkB Signaling Exacerbates Early Post‐Stroke Seizure Susceptibility

**DOI:** 10.1002/cns.70795

**Published:** 2026-02-13

**Authors:** Jing Zhou, Benjamin H. Wang, Jiangning Yu, Guoxiang Wang, Jingyi Cai, Mohan Yu, Kehua Chen, Li Wan, Xu Liu, Zhigang Yang, Yulong Wang, Yun Wang

**Affiliations:** ^1^ Department of Neurosurgery and Neurology, Institutes of Brain Science, State Key Laboratory of Medical Neurobiology and MOE Frontiers Center for Brain Science, Institute of Biological Science, Zhongshan Hospital Fudan University Shanghai China; ^2^ Hangzhou Linping District Integrated Traditional Chinese and Western Medicine Hospital Hangzhou Zhejiang China; ^3^ Department of Rehabilitation the First Affiliated Hospital of Shenzhen University/Shenzhen Second People's Hospital Shenzhen China

**Keywords:** BDNF, chloride homeostasis, ischemic stroke, microglia, potassium‐chloride cotransporter 2, seizures, TrkB

## Abstract

**Background:**

Post‐stroke seizures are a common and debilitating complication with limited therapeutic options, underscoring the need to identify novel molecular targets. Disruption of chloride homeostasis via impaired potassium chloride cotransporter 2 (KCC2) activity is a key driver of neuronal hyperexcitability. While microglia are a predominant source of brain‐derived neurotrophic factor (BDNF) in the acute phase after brain injury, the role of microglial BDNF and its signaling in KCC2 dysregulation and early post‐stroke seizure susceptibility remain poorly defined.

**Methods:**

Using a middle cerebral artery occlusion‐reperfusion (MCAO‐R) mouse model and oxygen–glucose deprivation/reoxygenation (OGD/R) in hippocampal neurons, we assessed KCC2 function, neuronal excitability, and seizure susceptibility. Pharmacological tools, including the microglial inhibitor minocycline, the TrkB antagonist K252a, the loop diuretic furosemide (FUR), repurposed here as a KCC2‐stabilizing agent, and the KCC2 activator CLP290, were employed. Techniques included immunofluorescence, Western blotting, patch‐clamp electrophysiology, electroencephalography (EEG), and behavioral seizure assessment.

**Results:**

MCAO‐R and OGD/R significantly reduced membrane KCC2 expression, leading to a depolarizing shift in the GABA equilibrium potentials (*E*_*GABA*_), diminished GABAergic inhibition, and increased neuronal excitability. Preventing KCC2 downregulation with FUR or CLP290 suppressed epileptiform activity in vitro and increased seizure thresholds in vivo. Ischemia induced robust microglial activation and increased BDNF release. Pharmacological inhibition of microglia (minocycline) or TrkB (K252a) effectively restored KCC2 expression, normalized *E*_*GABA*_, and reduced post‐stroke seizure severity.

**Conclusion:**

Our findings identify microglia‐derived BDNF/TrkB signaling as a critical upstream pathway mediating KCC2 dysfunction in early post‐stroke seizure. Targeting this axis by inhibiting microglial activation, blocking TrkB, or directly enhancing KCC2 function with activators like CLP290 represents a promising therapeutic strategy for stroke‐related epilepsy.

## Graphical abstract

Ischemic stroke induces microglial activation and BDNF–TrkB signaling, leading to KCC2 downregulation and seizure susceptibility. Pharmacological inhibition of microglia or TrkB and restoration of KCC2 function with the chloride extrusion enhancer CLP290 attenuate post‐ischemic seizures. These data identify KCC2 as a promising therapeutic target for post‐stroke seizures.

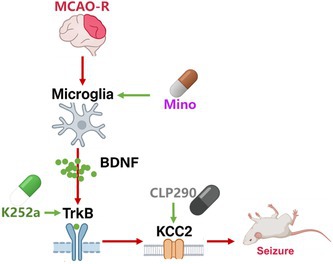

## 1 Introduction

Stroke is a leading cause of acquired seizures in adults, and seizures are among the most common neurological sequelae after stroke [1]. Seizures following ischemic stroke can prolong hospital stay and increase mortality [2, 3]. A fundamental factor in epileptogenesis is an imbalance between excitation and inhibition within neuronal networks in the brain [4]. Chloride transporters play key roles in modulating neuronal excitability and seizure thresholds by regulating neuronal chloride homeostasis [5, 6], and thereby determine the efficacy of GABA_A_ receptor (GABA_A_R) mediated inhibition in the brain.

KCC2 is predominantly expressed in neurons and functions to maintain low intracellular chloride concentrations [6]. This gradient allows GABA_A_R activation to produce hyperpolarizing and inhibitory responses [7, 8]. Downregulation of KCC2 expression disrupts this gradient, leading to reduced GABA‐mediated inhibition and can even paradoxically render GABAergic signaling depolarizing and excitatory via chloride influx [9]. Numerous studies have shown that neonatal cerebral hypoxic injury alters membrane KCC2 expression and function [10, 11], thereby affecting the progression of neonatal epileptogenesis. However, in adults, although only a few studies have demonstrated a significant neuron‐specific downregulation of KCC2 following focal cerebral ischemia [12, 13, 14], whether alterations in KCC2 expression following hypoxic injury in adults are related to post‐stroke seizures remains unexplored.

Following brain injury, microglia rapidly become activated in response to neuronal death and inflammation and secrete proinflammatory cytokines (TNF‐α, IL‐1β) and neurotrophins, including BDNF [15, 16]. Multiple studies have shown that microglia are the earliest and most robust responders in the acute post‐ischemic window (≤ 24 h) [17, 18, 19, 20], and a major source of BDNF after CNS injury. BDNF signaling has been implicated in epileptogenesis through increased neuronal hyperexcitability, aberrant network synchronization, and seizure activity [21, 22]. BDNF and its high‐affinity receptor TrkB can downregulate neuronal KCC2 expression [22, 23], especially during the acute phase of injury [24]. These considerations provide a clear rationale to focus on microglia‐derived BDNF in the early post‐stroke period as a potential upstream driver of KCC2 dysregulation and increased seizure susceptibility.

In this study, using the MCAO‐R mouse model, we aimed to determine whether neuronal KCC2 expression is altered during the acute phase after ischemic stroke and whether such changes are associated with post‐stroke seizure susceptibility. We further investigated whether MCAO‐R induces excessive microglial activation and engages BDNF/TrkB signaling, thereby contributing to KCC2 downregulation, reduced GABAergic inhibition, enhanced neuronal excitability, and epileptiform activity.

## 2 Materials and Methods

### 2.1 Animal Experiments

All animal procedures complied with institutional guidelines for animal care and use, adhered to ARRIVE recommendations, and were approved by the Fudan University Animal Ethics Committee (No. 20170223‐102) and the Shenzhen Second People's Hospital Animal Ethics Committee (No. 202200106). In vitro, timed‐pregnant Sprague–Dawley rats at gestational Day 17–18 were used. Hippocampi were dissected from fetuses and used to prepare primary hippocampal neuron cultures for subsequent OGD/R experiments. Adult male C57BL/6J mice (6–8 weeks, 20–25 g) were used for all in vivo experiments. All experimental animals were purchased from Shanghai SLAC Laboratory Animal Co. Ltd. (Chinese Academy of Sciences, Shanghai, China).

### 2.2 Middle Cerebral Artery Occlusion and Reperfusion

The MCAO‐R approach was used to induce cerebral ischemia as previously outlined [25]. Mice were anesthetized with 1.5%–2% isoflurane in oxygen. A central neck incision was performed, exposing the left common carotid artery (CCA), external carotid artery (ECA), and internal carotid artery (ICA). A 5–0 nylon monofilament suture with a rounded tip was inserted through the left CCA into the ICA until slight resistance indicated that the suture had reached the middle cerebral artery (MCA). Body temperature was maintained at 37°C ± 0.5°C using a heating pad. Following a designated 60‐min period of occlusion, the suture was removed to facilitate reperfusion.

To verify occlusion and reperfusion of the MCA, cortical blood flow was monitored using laser speckle contrast imaging (RWD Life Sciences, Shenzhen, China) [26]. Occlusion was considered successful when cortical perfusion decreased by > 70% of baseline and reperfusion when it recovered to > 50% of baseline. Following a scalp incision, the mouse skull was completely exposed and kept moist with saline. The MCA territory in the target hemisphere was then identified, and average cerebral blood flow readings were acquired.

### 2.3 Modified Neurological Severity Score Assessment and TTC Staining

Neurological deficits were assessed using the mouse modified neurological severity score (mNSS) [27], which ranges from 0 (normal) to 14 (maximal deficit) and can be stratified into mild (1–4), moderate (5–9), and severe (10–14) impairment [28]. In this study, only mice with moderate to severe deficits (mNSS 5–14) after MCAO‐R were included in subsequent analyses to ensure a consistent level of injury severity.

TTC staining was performed to verify infarct formation 23 h after reperfusion, as previously described [29, 30]. Brains were quickly removed and coronally sectioned into 2‐mm slices, then incubated in 2% TTC solution (Sigma‐Aldrich) in PBS (pH 7.4) at 37°C for 15 min in the dark, followed by fixation in 4% paraformaldehyde overnight at 4°C. Non‐infarcted tissue appeared red, whereas infarcted regions remained white.

Images were digitized using a flatbed scanner and analyzed with ImageJ (NIH, USA). Infarct volume was calculated as the sum of infarct areas across all sections, corrected for edema using the standard formula. TTC together with LSCI and mNSS scoring was used to verify successful MCAO‐R induction (Figure S1).

### 2.4 Pharmacological Inhibitor Preparation

For furosemide (FUR) administration, a guide cannula was pre‐implanted into the lateral ventricle 1 week before MCAO‐R surgery. A guide cannula was implanted into the right lateral ventricle (0.3 mm posterior to bregma, 1.3 mm lateral to the midline, 4.0 mm below the skull surface) according to a mouse brain stereotaxic atlas. FUR (5 μM, 2.5 μL, i.c.v.) was used at a low dose previously shown to prevent convulsant‐induced KCC2 downregulation without affecting NKCC1 activity, thus acting as a stabilizer of KCC2‐dependent inhibition rather than a broad cotransporter blocker [31]. K252a (a kinase inhibitor commonly used to block TrkB autophosphorylation) [32, 33], minocycline (a widely used inhibitor of microglial activation with pleiotropic actions) [34], and CLP290 (a KCC2 activator) [35, 36] were administered intraperitoneally 30 min before the MCAO‐R surgery. Doses (K252a 25 μg/kg [33], minocycline 90 mg/kg [34], CLP290 35 mg/kg [35]) were selected based on previous studies demonstrating efficacy in ischemia or seizure models. FUR was purchased from Sigma‐Aldrich, and minocycline, K252a, and CLP290 were from MedChemExpress. All drugs were first dissolved in DMSO and then diluted in saline, resulting in a final DMSO concentration of 0.1% in each treatment solution.

### 2.5 Culture of Primary Hippocampal Neurons

Timed‐pregnant Sprague–Dawley rats at gestational Day 17–18 were anesthetized with 25% urethane (0.4 mL/kg, i.p.), and fetuses were rapidly removed by cesarean section. Hippocampi were dissected, and blood vessels were cleared [37, 38]. Hippocampal tissue was digested with preheated trypsin for 15 min with shaking, then halted using D/F12 medium with serum. The tissue was washed (1000 rpm, 1 min) with D/F12 enzyme‐free wash solution, and the supernatant was gently aspirated. The tissue was finally resuspended in 10 mL D/F12 medium and gently triturated to obtain a single‐cell suspension. Cells were cultured in 24‐well plates on 12 mm coverslips, adding D/F12 medium to 1.6 mL final volume, and placed in a 37°C, 5% CO_2_ humidified incubator. The medium was exchanged with NB27 after 24 h and every 3 days thereafter, supplemented with 2 μM cytosine arabinoside once glial cell confluency was confirmed after 6 days.

### 2.6 Oxygen–Glucose Deprivation/Reoxygenation

OGD was used as a standard in vitro model of ischemia involving glucose and oxygen deprivation [39]. Primary hippocampal neurons at DIV15 were subjected to OGD followed by reoxygenation. Cells were washed in a glucose‐free medium and placed in an anoxic chamber with 95% N_2_ and 5% CO_2_ for 30 min to achieve 0.5%–1% O_2_. Subsequently, they were returned to normal glucose and oxygen conditions (95% air, 5% CO_2_) in a standard cell culture chamber for 4 h of reoxygenation. Primary cultured hippocampal neurons at DIV15 were subjected to various treatments, and cells were randomly assigned to four groups: (i) Control: 0.1% DMSO without OGD/R; (ii) OGD/R: 0.1% DMSO + OGD/R; (iii) FUR + OGD/R: 100 μM FUR [40] present during OGD; and (iv) K252a + OGD/R: 100 nM K252a [41] present during OGD.

### 2.7 Brain Slice Preparation

An NMDG‐based cutting solution was prepared containing (in mM): 92 NMDG, 2.5 KCl, 1.0 NaH_2_PO_4_, 20 HEPES, 30 NaHCO_3_, 25 D‐glucose, 5 ascorbic acid, 5 pyruvic acid, 2 thiourea, 1.3 MgSO_4_, and 2.5 CaCl_2_ (pH 7.3–7.4; ~300 mOsm). The solution was stored at 4°C until use. Brain extraction and sectioning were performed in ice‐cold NMDG solution, and all instruments were pre‐chilled on ice to minimize tissue damage. Artificial cerebrospinal fluid (ACSF) was prepared on the day of electrophysiological experiments and contained (in mM): 119 NaCl, 2.5 KCl, 2.5 CaCl_2_, 1.3 MgSO_4_, 1.0 NaH_2_PO_4_, 26.2 NaHCO_3_, and 11 D‐glucose (pH 7.3; ~300 mOsm).

For slice preparation, mice were anesthetized with sodium pentobarbital (60 mg/kg, i.p.) and decapitated. Brains were rapidly removed and glued to an agar block in the slicing chamber. Coronal brain slices (300 μm) containing the hippocampus were cut in ice‐cold, carbogenated (95% O_2_/5% CO_2_) NMDG solution using a vibrating microtome (VT1200S, Leica Instruments, Germany). Slices were then incubated at 34°C for 30 min before being transferred to ACSF at room temperature (24°C ± 1°C) for at least 1 h before electrophysiological recording. Slices from the ipsilateral (ischemic) and contralateral hemispheres of MCAO‐R mice were incubated in separate chambers.

### 2.8 Electrophysiology Recordings

Whole‐cell patch‐clamp recordings were obtained from hippocampal CA1 pyramidal neurons in acute slices at 23 h after MCAO‐R and from primary hippocampal neurons using established methods [42, 43]. Signals were recorded with a Multiclamp 700B amplifier, digitized with a Digidata 1440A interface, and acquired using pCLAMP software.

For measurements of the GABAergic reversal potential (*E*_*GABA*_), CA1 pyramidal neurons were voltage‐clamped with small‐tip pipettes (10–15 MΩ) filled with a low‐chloride K‐gluconate–based internal solution. *E*_*GABA*_ was measured with small‐tip whole‐cell recordings as a functional proxy for neuronal [Cl^−^]_i_ and KCC2 function [42, 43]. Cells were held at potentials between −80 and −30 mV, and GABA_A_R–mediated currents were evoked by brief pressure application of GABA (250 μM). DNQX (20 μM), D‐AP5 (50 μM), and tetrodotoxin (TTX, 1 μM) were included to block ionotropic glutamate receptors and action potentials. *E*_*GABA*_ was determined as the x‐intercept of the current–voltage (I–V) relationship.

Miniature inhibitory postsynaptic currents (mIPSCs) were recorded with 3–5 MΩ pipettes containing a Cs‐based internal solution with QX‐314. During mIPSC recordings, DNQX (20 μM), D‐AP5 (50 μM), and TTX (1 μM) were present in the ACSF, and neurons were voltage‐clamped at 0 mV. Synaptic events were detected and analyzed offline using predefined thresholds by an experimenter blinded to treatment group.

For cultured hippocampal neurons, pharmacological pretreatments (FUR 100 μM, K252a 100 nM, or vehicle) were initiated at DIV 15, followed by 30 min of OGD and 4 h of reoxygenation before recording, following published protocols [44, 45, 46]. Epileptiform activity was recorded in conventional whole‐cell current‐clamp mode using a K‐gluconate–based internal solution while holding the membrane potential near −70 mV [44, 45, 46]. Epileptiform discharges were defined as depolarizing events ≥ 10 mV in amplitude lasting ≥ 300 ms with ≥ 5 action potentials riding on the depolarizing envelope, and neurons were classified as “bursting” if at least two such bursts occurred within a 10‐min recording period [44, 46].

### 2.9 Behavioral Assessment of Seizure Susceptibility

Seizure susceptibility was evaluated using a pentylenetetrazol (PTZ)‐induced seizure test, in which PTZ, a GABA_A_ receptor antagonist, was administered intraperitoneally 23 h after MCAO‐R [47]. Behavioral testing was performed 23 h after MCAO‐R by an investigator blinded to the treatment group. PTZ was purchased from Sigma‐Aldrich (Cat#P6500) and prepared fresh before each seizure induction. Before starting the PTZ injection, the mice were transferred to a transparent cage and allowed to adapt for at least 30 min. PTZ was administered intraperitoneally to mice starting with an initial dose of 40 mg/kg. The dose was then increased by 10 mg/kg every 10 min until the mice exhibited Racine grade IV‐V seizures or the cumulative PTZ dose reached a maximum of 70 mg/kg. The behavioral performance of seizures was evaluated according to the classification method of Racine [31, 48]. The Racine scoring system was used to assess the severity of seizures [48, 49], with scores ranging from 0 (no seizure activity) to 5 (tonic–clonic seizures). Racine I: Facial twitching accompanied by chewing, blinking, staring, glowering or erect tail; Racine II: Head nodding, unilateral limb twitching or dorsal tonus, spinning, shaking; Racine III: Whole body shaking and twitching or tonic, tail erect; Racine IV: Twitching of both forelimbs while standing, falling back; Racine V: Loss of balance and falling to one side, continued running, jumping or death.

### 2.10 Electroencephalographic Recordings

Male C57BL/6 mice were anesthetized with isoflurane and placed in a stereotaxic frame. After exposing the skull, small burr holes were drilled to accommodate stainless‐steel screw electrodes. A reference electrode was placed over the forehead (AP −1.5 mm, ML −1.5 mm), and a recording electrode targeted the left hippocampus (AP −1.8 mm, ML −1.4 mm). Electrodes were secured with dental cement. After surgery, mice received saline/glucose support and underwent MCAO‐R 5–7 days later. Drugs (minocycline, K252a, CLP290, or vehicle) were administered before MCAO‐R as described. At 23 h after MCAO‐R, mice were transferred to the recording chamber and habituated for ≥ 30 min. A 30‐min baseline EEG was recorded, followed by PTZ (40 mg/kg, i.p.) to induce seizures, with simultaneous EEG and behavioral monitoring. Signals were amplified (×1000), band‐pass filtered (5–500 Hz) using a NeuroLog System (Digitimer, UK), digitized at 1 kHz via CED Micro1401, and stored with Spike2 software (Cambridge Electronic Design, UK). Spontaneous epileptiform discharges were defined as sharp‐wave events with amplitude > 2× baseline SD and duration ≥ 20 ms occurring at > 1 Hz. All events were independently adjudicated by two investigators blinded to group assignment.

### 2.11 Immunofluorescence Analysis

To analyze KCC2 expression and microglial activation, we collected brain tissue 23 h after MCAO‐R. Brains were fixed in 4% paraformaldehyde for 24 h and then cryoprotected in 15% and 30% sucrose solutions. Subsequently, the fixed brains were mounted in OCT gels and stored at −80°C. Coronal sections (30 μm) containing the hippocampus were cut on a cryostat. These slices underwent thorough rinsing with PBS, followed by permeabilization and incubation with 0.2% Triton‐X 100 and 10% NDS (donkey serum) for 2 h at room temperature. Subsequently, the sections were exposed to primary antibodies (rabbit anti‐KCC2 at 1:300, #07–432 from Millipore, rabbit anti‐Iba1 at 1:500, #17198 from Cell Signaling Technology, guinea pig anti‐NeuN at 1:500, #ABN90 from Millipore) diluted in 10% NDS overnight at 4°C. After three PBS rinses, the sections were incubated with secondary antibodies (Alexa Fluor 488 anti‐Rabbit IgG at 1:500, Alexa Fluor 647 anti‐Guinea IgG at 1:500) diluted in 10% NDS and incubated in darkness for 2 h at room temperature. DAPI staining (1:1000) served as a tracer for 15 min, and application of an anti‐quenching reagent. Imaging was performed using confocal scanning microscopy with a 25× water immersion objective from Nikon (A1R). ImageJ software was utilized for fluorescence quantification of KCC2/NeuN fluorescence intensity (gray values). The fluorescence intensity of KCC2 in sham mice was set at 100%.

Quantitative analysis of Iba1^+^ and GFAP^+^ cells was performed using ImageJ. The following primary antibodies were used: rabbit anti‐Iba1 (1:500; Wako, Cat# 019–19,741) and rabbit anti‐GFAP (1:500; Abcam, Cat# ab7260). The number of positive cells per mm^2^ and mean fluorescence intensity were calculated in the peri‐infarct hippocampal CA1 region.

Coverslips with primary cultured hippocampal neurons were fixed in 1% paraformaldehyde for 15 min, washed with PBS for 5 min, and permeabilized with 5% donkey serum and 0.1% Triton for 1 h. Cell sheets were incubated overnight at 4°C with primary antibodies against KCC2 (1:300, Millipore #07‐432) and NeuN (1:500, Millipore #ABN90). The antibody sources were the same as those used for brain sections. Cell sheets were retrieved to room temperature for 30 min the next day, followed by three 5‐min PBS washes. Fluorescent secondary antibodies (1:500) labeled with Alexa Fluor 488 for KCC2 and Alexa Fluor 647 for NeuN were applied, and the sheets were incubated under light‐avoiding conditions for 1.5 h. The cell sheets were stained with DAPI for 10 min. Imaging was performed using an Olympus BX5 laser confocal microscope, with 3 regions per cell sheet examined. For each coverslip, 5–8 neurons were selected, and the average membrane‐associated KCC2 fluorescence intensity was quantified. The KCC2 fluorescence intensity of neurons in the control group from the same batch was designated as 100%, and the percentage of KCC2 expression in each group relative to the control group was calculated to assess differences in KCC2 expression on the membrane of hippocampal neurons across groups.

### 2.12 Western Blot Analysis

To assess membrane KCC2 protein expression levels, Western blot analysis was conducted on whole hippocampal tissue homogenates extracted from the ischemic injury side using a membrane protein extraction kit (Thermo Fisher, #89842). Tissues were homogenized in RIPA buffer supplemented with protease and phosphorylase inhibitors. The homogenate was centrifuged at 700 *g* for 10 min at 4°C, and the supernatant was carefully collected and centrifuged again at 10,000 *g* for 30 min. The resulting supernatant contained cytoplasmic proteins, whereas the pellet was enriched for plasma membrane proteins. The membrane protein pellet was resuspended in PBST containing SDS and heated at 45°C for 45 min to facilitate solubilization and inactivate enzymes. Protein concentration was determined using the BCA Protein Assay Kit (Thermo Fisher, #23227). Equal protein amounts were separated by 10% SDS‐PAGE and transferred electrophoretically onto PVDF membranes (0.45 μm, Millipore). Membranes were incubated overnight at 4°C with specific primary antibodies: rabbit anti‐KCC2 (1:1000, Millipore #07‐432), mouse anti‐GAPDH (1:2000, Abcam # ab263962), guinea pig anti‐NeuN (1:500, Millipore #ABN90), and mouse anti‐BDNF (1:1000, Abcam # ab203573) in 5% skim milk‐TBST buffer. This was followed by incubation with goat anti‐rabbit, anti‐mouse, or anti‐guinea pig (Abcam #ab6908) secondary antibodies (1:5000). KCC2 expression was normalized to GAPDH and NeuN levels on the same blot. Protein bands were visualized using an ECL detection system (Protein Sample, USA), and quantification was performed using ImageJ software. Values are expressed as a percentage of the sham control. All Western blot quantifications were performed on 3–4 biological replicates per group as stated in Results.

### 2.13 Statistical Analysis

All statistical analyses were performed using GraphPad Prism 8.0 and IBM SPSS Statistics 25.0. Data are presented as mean ± standard deviation (SD). The sample size N refers to the number of animals per group, while n represents the number of cells or neurons analyzed. Sample sizes were determined by a priori power analysis (G*Power 3.1) based on pilot data and previously reported effect sizes in similar models of ischemia [12] and seizure susceptibility [36, 50]. Assuming a medium effect size (*f* = 0.4), *α* = 0.05, and desired power (1 − β) = 0.8, the analysis indicated a minimum of *N* = 4–13 animals per group for in vivo behavioral and histological assays, and *n* = 15–24 neurons per group for electrophysiological recordings. All experimental groups met or exceeded these thresholds.

For comparisons between two independent groups, unpaired Student's *t*‐tests were used for normally distributed continuous variables. One‐way ANOVA followed by Tukey's post hoc test was applied for comparisons across three or more groups. When data violated assumptions of normality or homogeneity of variance, nonparametric Kruskal‐Wallis tests with Dunn's post hoc correction were used. Categorical data, including the proportion of neurons exhibiting epileptiform bursting activity in vitro and the incidence of Racine stage IV–V seizures in vivo, were analyzed using the Chi‐square test (*χ* ^2^ test).

Western blot and immunofluorescence quantification data are expressed as a percentage of control (sham) group values to allow normalization across blots or imaging sessions. Fluorescence intensity measurements were conducted using ImageJ software, with background subtraction and blinded analysis to minimize bias. All patch‐clamp experiments included appropriate controls, and recordings were performed under blinded conditions whenever feasible. While the experimenter performing drug administration was not blinded, all behavioral scoring and data analysis were performed by investigators blinded to the experimental group. Statistical significance was defined as *p* < 0.05. Asterisks denote: *p < 0.05, **p < 0.01, ***p < 0.001 compared to the sham or control group; pound signs denote: #*p* < 0.05, ##*p* < 0.01, ###*p* < 0.001 compared to the MCAO‐R or OGD/R group.

## 3 Results

### 3.1 Downregulation of KCC2 After MCAO‐R Is Associated With Increased Seizure Susceptibility in Mice

**FIGURE 1:**
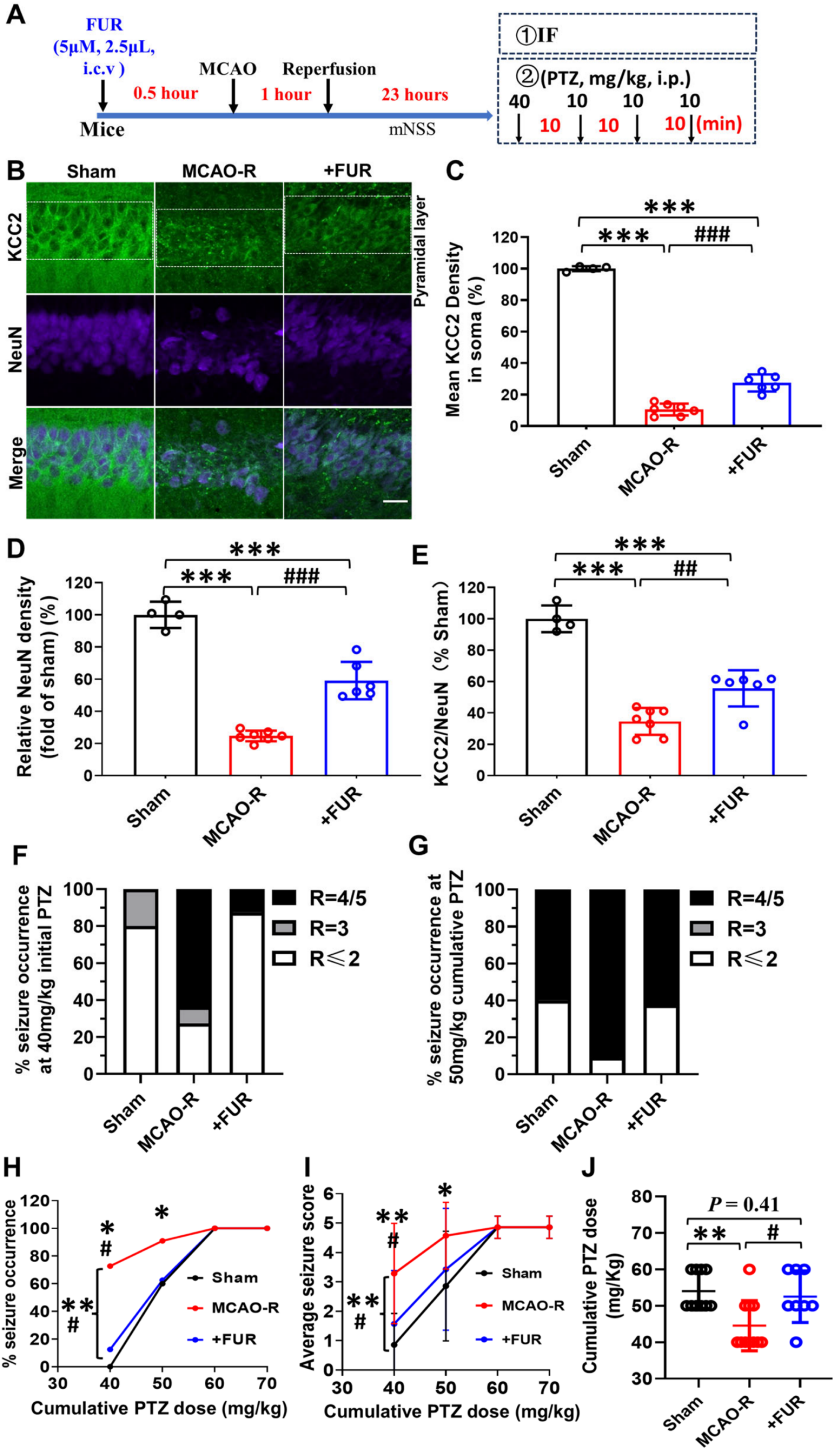
Prevention of KCC2 downregulation after MCAO‐R mitigates seizure occurrence and severity. (A) Experimental protocols for immunofluorescence and PTZ in mice. (B) Confocal images depicting KCC2 immunostaining in hippocampal CA1 neurons from MCAO‐R mice with or without FUR treatment, compared to sham. The scale bars measure 50 μm. (C) Quantitative assessment of KCC2 labeling density in cells of the pyramidal layer labeling region of hippocampal CA1 neurons (Sham, *N* = 4; MCAO‐R, *N* = 7; MCAO‐*R* + FUR, *N* = 6). (D) Quantitative analysis of NeuN labeling density in cells of the pyramidal layer labeling region of hippocampal CA1 neurons. (E) Quantitative evaluation of membrane KCC2 labeling density in fluorescently labeled cells relative to NeuN. (F, G) Distribution of mice across different seizure scores at 40 mg/kg (F) and 50 mg/kg (G) cumulative PTZ dosage. (H, I) Line graphs demonstrate the impact of preventing the KCC2 downregulation on the percentage of MCAO‐R mice displaying Racine IV‐V seizure behaviors (H) and the average seizure score (I) at each cumulative PTZ dose. (J) The cumulative PTZ dose elicited Racine IV‐V epileptic behaviors. (Sham: *N* = 9, MCAO‐R: *N* = 10, FUR+MCAO‐R: *N* = 7.) *p < 0.05, **p < 0.01, and ***p < 0.001 in comparison to the sham group. ^#^ *p <* 0.05, ^##^ *p* < 0.01, and ^###^ *p* < 0.001 compared to MCAO‐R.

The cumulative PTZ injection protocol was used to test whether MCAO‐R increases seizure susceptibility and whether preventing KCC2 downregulation with the loop diuretic FUR (5 μM, 2.5 μL, i.c.v.) alters this effect (Figure 1A). In the following, we use the term “KCC2 downregulation” to refer to reduced KCC2 protein expression (in particular, membrane‐localized KCC2), and “KCC2 dysfunction” to refer to functional impairments such as a depolarizing shift in *E*_*GABA*_ and weakened GABA_A_R‐mediated inhibition.

In the MCAO‐R group, the KCC2 immunolabeling density was significantly reduced in the hippocampus region of the CA1 pyramidal cell layer (Figure 1B). The mean KCC2 immuno‐signal density in the MCAO‐R group was reduced to 10.6% ± 3.7% of the sham group (*F*_(2,14)_ = 621.80, followed by Tukey's test, *p* < 0.001; Figure 1B,C). FUR pretreatment significantly restored KCC2 labeling to 27.4% ± 5.4% of sham levels (*p* < 0.001, Figure 1B,C). NeuN protein expression in the pyramidal cell layer of MCAO‐R mice significantly decreased to 25.6% of the sham group (*F*_(2,14)_ = 108.8, *p* < 0.001, Figure 1D), suggesting substantial neuronal loss during ischemic insult. FUR pretreatment in MCAO‐R significantly restored NeuN immunoreactivity in the CA1 pyramidal cell layer to 59.1% ± 11.6% of the sham group (Figure 1D), indicating partial preservation of neuronal survival. To better represent neuronal KCC2 expression, KCC2 levels were normalized to NeuN expression [12]. The normalized KCC2: NeuN ratio still showed a significant downregulation after MCAO‐R, and this was significantly attenuated by FUR pretreatment (KCC2: NeuN ratio in MCAO‐R: 34.6% ± 8.6% of sham vs. FUR+MCAO‐R: 55.7% ± 11.5% of sham, *F*_(2,14)_ = 57.73, *p* < 0.01, Figure 1E). These results indicate that ischemic insult is associated with marked KCC2 downregulation in CA1 pyramidal neurons and that FUR partially preserves neuronal KCC2 expression.

We next examined whether preventing KCC2 downregulation with FUR affects seizure susceptibility in MCAO‐R mice. In the MCAO‐R group, seizure susceptibility was increased relative to sham, as evidenced by higher incidence and severity of PTZ‐induced seizures. Within 10 min of an initial subthreshold PTZ dose (40 mg/kg, i.p.), most of the sham mice (8 of 10) exhibited mild Racine I‐II seizure behaviors, with few (2 of 10) reaching Racine III, while more than half of the MCAO‐R mice (7 of 11) experienced severe Racine IV‐V seizures, one MCAO‐R mouse exhibited Racine III, and the remainder showed Racine II‐III (Figure 1F). At 50 mg/kg PTZ, a higher percentage of MCAO‐R mice exhibited Racine IV–V seizures compared with sham (Sham: 60% vs. MCAO‐R: 91%, *p* = 0.030, Figure 1G). Average seizure scores in the MCAO‐R group were significantly higher than those in the sham mice across cumulative PTZ doses (40 mg/kg: 0.8 ± 1.2 vs. 3.3 ± 1.4, *p* < 0.001; 50 mg/kg: 2.9 ± 2.0 vs. 4.4 ± 1.0, *p* = 0.045, Figure 1H). FUR pretreatment reduced seizure severity in MCAO‐R mice. It significantly decreased the incidence of Racine IV–V behaviors at 40 mg/kg PTZ (MCAO‐*R* + FUR: 13% vs. MCAO‐R: 87%, *χ* ^2^ = 6.80, *p* = 0.033, Figure 1F,G) and 50 mg/kg PTZ (MCAO‐R + FUR: 63% vs. MCAO‐R: 91%, *χ* ^2^ = 2.25, *p* = 0.134, Figure 1G,H), and lowered average seizure scores at 40 mg/kg PTZ (40 mg/kg: FUR + MCAO‐R: 1.4 ± 1.8, *N* = 8 vs. MCAO‐R: 3.3 ± 1.4, *N* = 11, *t*_(17)_ = 2.33, *p* = 0.030, Figure 1I). The cumulative PTZ doses required to induce Racine IV‐V seizures were significantly lower in MCAO‐R mice than those in the sham group (One way‐ANOVA *F*_(2,26)_ = 6.57, *p* < 0.001, followed by Tukey's test, sham: 54.0 ± 5.3 mg/kg, *N* = 10 vs. MCAO‐R: 44.6 ± 7.0 mg/kg, N = 11, *p* = 0.006, Figure 1J), whereas FUR pretreatment increased the cumulative PTZ dose (MCAO‐R vs. MCAO‐*R* + FUR: 52.5 ± 6.9 mg/kg, N = 8, *p* = 0.033, Figure 1J). Our findings suggest that blocking KCC2 downregulation with FUR reduces MCAO‐R‐enhanced seizure susceptibility in vivo.

### 3.2 Downregulation of mKCC2 After OGD/R Is Accompanied by Increased Epileptiform Bursts in Primary Cultured Hippocampal Neurons

**FIGURE 2:**
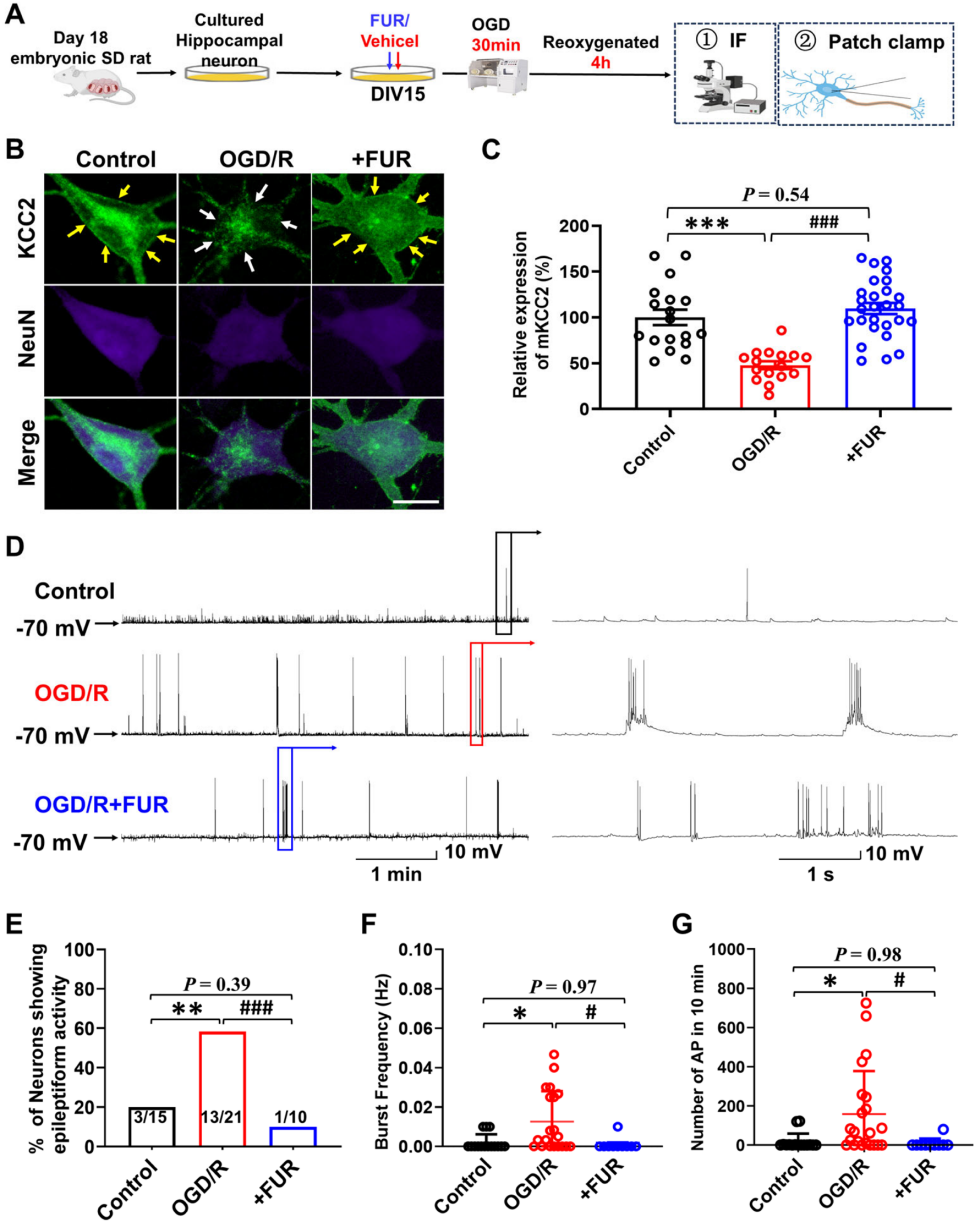
Mitigation of OGD/R‐induced KCC2 downregulation reduces the occurrence of epileptiform bursting activity in vitro. (A) Primary hippocampal neurons were cultured, FUR pre‐treated, OGD/R, immunofluorescence staining, and whole‐cell membrane clamp schematic. (B) Microscopic images illustrating KCC2 expression in cultured hippocampal neurons in the control group, OGD/R group, and OGD/*R* + FUR group. The green and blue‐violet signals represent KCC2 and NeuN immunostaining, respectively. The scale bar is 5 μm. (C) Quantitative assessment of membrane KCC2 in cultured hippocampal neurons (Control: *N* = 23 cells; OGD/R: *N* = 21; FUR+OGD/R: *N* = 28, all from *N* = 5 independent cultures). (D) Representative traces demonstrate the inhibition of epileptiform burst firing induced by OGD/R in cultured hippocampal neurons following FUR administration. (E–G) A significant reduction in the proportion of neurons exhibiting epileptiform bursting firing (E), bursting frequency (F), and the total number of APs over 10 min (G) in all neurons after FUR pretreatment. (Control: *N* = 15 cells; OGD/R: *N* = 21 cells; FUR+OGD/R: *N* = 10 cells, all from *N* = 3 cultures) *p < 0.05, **p < 0.01, and ***p < 0.001 compared to the control group. ^#^ *p* < 0.05 and ^###^ *p* < 0.001 compared to OGD/R group.

Given the prominent decrease in mKCC2 expression after MCAO‐R, we next examined whether OGD/R in primary hippocampal neurons produces similar changes in mKCC2 and epileptiform bursting in vitro (Figure 2A). The results also showed that OGD/R significantly decreased the mKCC2 expression to 47.8% ± 17.0% of the control group (*F*_(2,58)_ = 22.54, *p* < 0.001, Figure 2B,C), whereas FUR pretreatment protected against this decrease (109.7% ± 31.7% of control, post hoc Tukey's test, *p* = 0.54, Figure 2B,C).

Blocking mKCC2 downregulation by FUR pretreatment effectively inhibited the OGD/R‐induced bursting activity primarily. Compared with control and FUR‐pretreated neurons, OGD/R neurons showed a higher percentage of epileptiform bursts within 10 min (*χ* ^2^ test, Control: 20% vs. OGD/R: 61.9%, *p* < 0.01; OGD/R vs. OGD/*R* + FUR: 10.0%, *p* < 0.001, Figure 2D,E), increased frequency of bursts (*F*_(2,42)_ = 5.76, post hoc Tukey's test, Control: 0.002 ± 0.004 Hz, *n* = 15 vs. OGD/R: 0.013 ± 0.015 Hz, *n* = 21, *p* = 0.017; OGD/R vs. OGD/*R* + FUR: 0.001 ± 0.003 Hz, *n* = 10, *p* = 0.022, Figure 2F), and a higher total number of action potentials (APs) (F_(2,44)_ = 5.17, post hoc Tukey's test, Control: 16.7 ± 42.3, *n* = 10 vs. OGD/R: 158.1 ± 220.0, *n* = 21, *p* = 0.024; OGD/R vs. OGD/*R* + FUR: 8.0 ± 25.3, *n* = 10, *p* = 0.037, Figure 2G). These results show that mitigation of OGD/R‐induced KCC2 downregulation reduces the occurrence of epileptiform bursting activity in vitro.

### 3.3 KCC2 Downregulation Is Associated With GABA_A_R‐Mediated Depolarization in the Pyramidal Neurons

**FIGURE 3:**
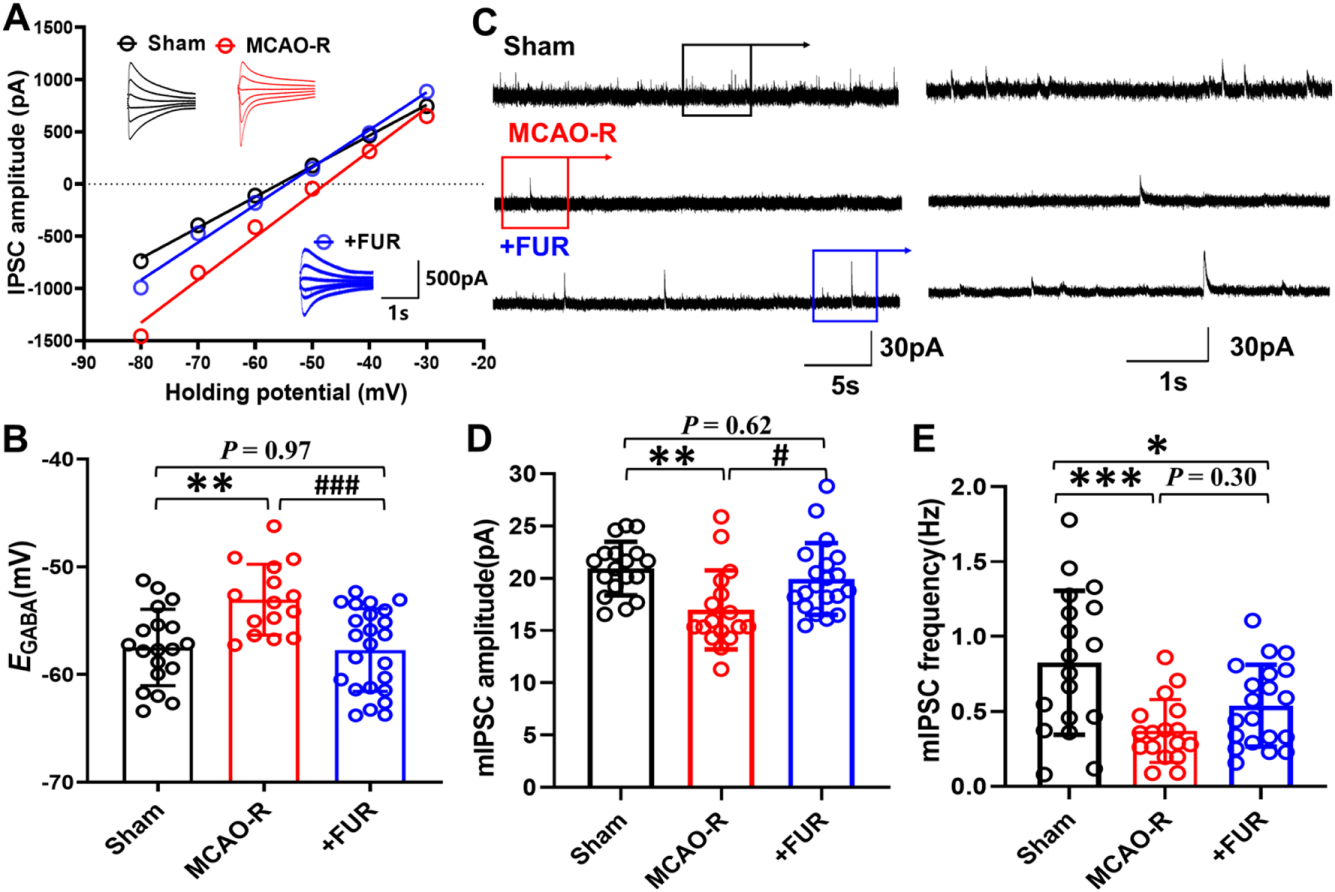
Preventing KCC2 down‐regulation reverses *E*_*GABA*_ depolarization, and the reduced efficiency of GABA_A_R function. (A) Small‐tip whole‐cell patch clamp recordings were performed to determine the *E*_*GABA*_ and I‐V plots were constructed. (B) Statistical analysis revealed a substantial positive shift in *E*_*GABA*_ post‐MCAO‐R, which was reversed by FUR pretreatment. (C–E) Patch clamp recordings using a low‐chloride electrode solution (C) indicated reductions in both the amplitude (D), where sham = 20.9 ± 2.7 pA, *N* = 4, *n* = 15, versus MCAO‐*R* = 16.9 ± 3.8 pA, *N* = 3, *n* = 17, and frequency (E) of mIPSCs, where sham = 1.0 ± 0.4 Hz, *n* = 15, versus MCAO‐*R* = 0.4 ± 0.2 Hz, *n* = 17, which are KCC2‐dependent, following MCAO‐R. However, FUR pretreatment (*N* = 4) only restored the mIPSCs amplitude (MCAO‐*R* + FUR: 19.9 ± 3.4 pA, *N* = 4, *n* = 20), not the frequency. *p < 0.05, **p < 0.01, and ***p < 0.001 compared to the control group; ^#^ *p* < 0.05 and ^###^ *p* < 0.001 compared to the MCAO‐R group.

Under normal physiological conditions, KCC2 maintains low intracellular chloride, so that GABA_A_R activation produces hyperpolarizing, inhibitory responses [9]. After MCAO‐R, reduced membrane KCC2 expression and a depolarizing shift in *E*_*GABA*_ weaken GABAergic inhibition and promote neuronal hyperexcitability. Twenty‐three hours following MCAO‐R, GABA‐induced responses were analyzed by plotting offline current–voltage curves. Significant depolarizing shifts in *E*_*GABA*_ continued to be recorded in the MCAO‐R group but not in the FUR pretreated group, as shown in the results (*F*_(2,55)_ = 8.94, post hoc Tukey's test, Sham: −57.5 ± 3.5 mV, *N* = 4, *n* = 19 vs. MCAO‐R: −53.1 ± 3.3 mV, *N* = 3, *n* = 15, *p* < 0.01; MCAO‐R vs. MCAO‐*R* + FUR: −57.7 ± 3.8 mV, *N* = 4, *n* = 24, *p* < 0.001, Figure 3A,B). These findings indicate that MCAO‐R induces KCC2 dysfunction, reflected by a depolarizing shift in *E*_*GABA*_, and that FUR pretreatment restores KCC2‐dependent chloride extrusion, normalizing *E*_*GABA*_ toward sham levels in line with its preservation of KCC2 expression.

The neural network's inhibitory synaptic transmission efficiency was evaluated through the measurement of miniature inhibitory postsynaptic currents (mIPSCs) via whole‐cell patch clamp methodology. To maintain the integrity of KCC2's role in Cl^−^transport and prevent interference from elevated Cl^−^ levels in the electrode solution, a low Cl^−^ solution was employed in this study [51]. Neurons were held at 0 mV to elicit upward mIPSCs, and the results demonstrated a reduction in both the amplitude (*p* < 0.01, Figure 3C,D) and frequency (*p* < 0.01, Figure 3C,E) of mIPSCs in the MCAO‐R group compared to the sham group. Furthermore, the blockade of KCC2 downregulation with FUR reversed the reduction of mIPSCs amplitude observed in MCAO‐R (*F*_(2,52)_ = 6.80, MCAO‐*R* + FUR: 19.9 ± 3.4 pA, *N* = 4, *n* = 20 vs. MCAO‐R: 16.9 ± 3.8 pA, *N* = 3, *n* = 17, *p* = 0.024, Figure 3C,D), but did not affect the frequency (*F*_(2,52)_ = 8.02, MCAO‐*R* + FUR: 0.5 ± 0.3 Hz, *n* = 20 vs. MCAO‐R: 0.4 ± 0.2 Hz, *n* = 17, *p* = 0.30, Figure 3C,E). Given that the mIPSCs amplitude reflects the efficiency of GABA_A_R function, which relies on mKCC2 in adult neurons, these findings indicate that MCAO‐R‐induced reduction in mKCC2 impairs GABA_A_R efficiency, and prevention of KCC2 downregulation can block KCC2 functional defects and GABA_A_R‐mediated depolarization, which was reflected by changes in the *E*_*GABA*_ and mIPSCs amplitudes.

### 3.4 MCAO‐R Induces KCC2 Downregulation and Dysfunction via Microglial Activation

**FIGURE 4:**
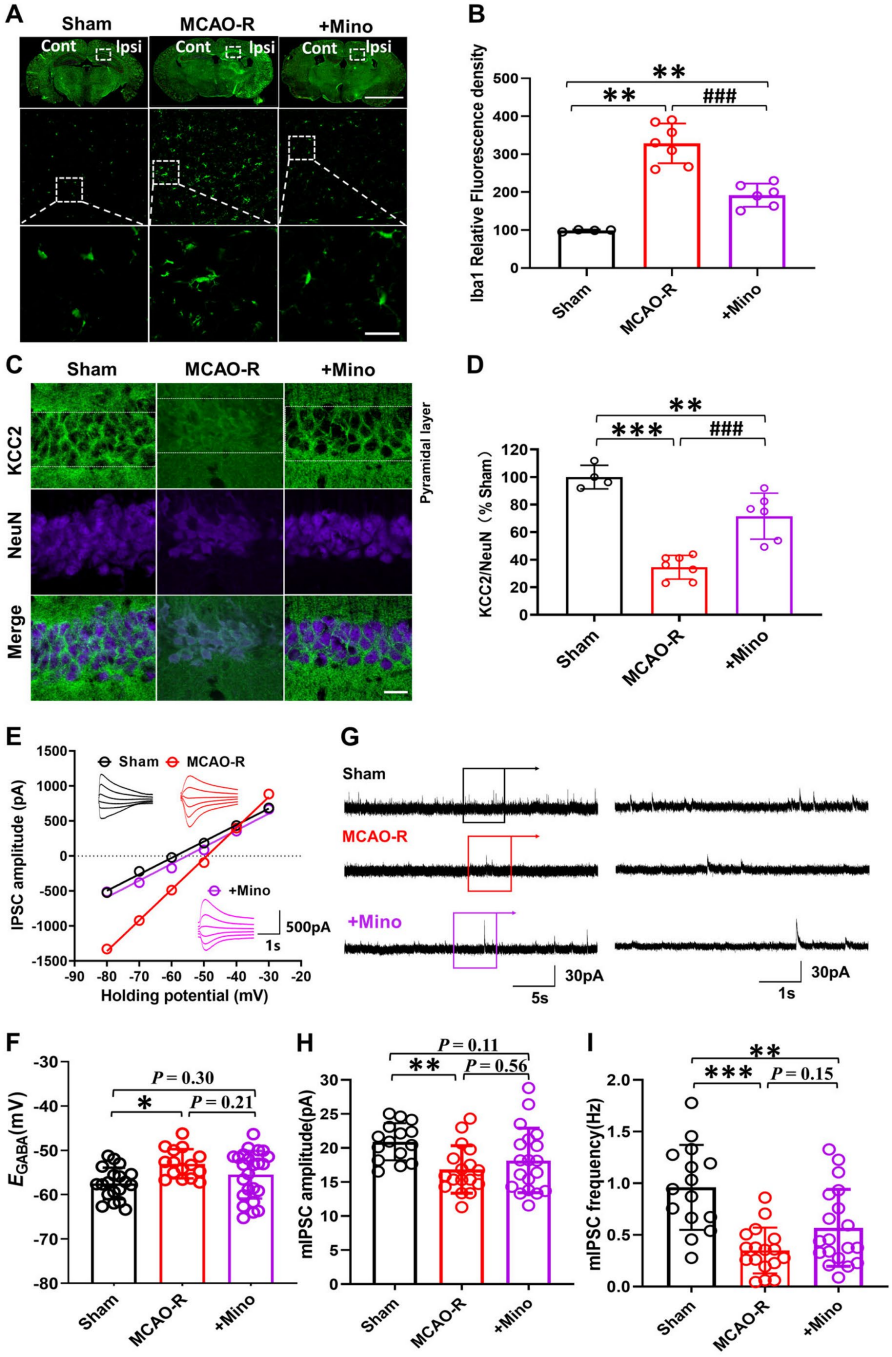
Suppression of the microglial activation ameliorates the KCC2 downregulation and dysfunction after hypoxia injury. (A) Representative immunofluorescence images of the hippocampal CA1 region in sham, MCAO‐R, or MCAO‐R pretreatment with minocycline. Scale bars: 5 mm and 50 μm. (B) Evaluation of Iba1 expression in hippocampal CA1 region neurons quantitatively (Sham: *N* = 4, MCAO‐R: *N* = 7, MCAO‐*R* + Mino: *N* = 6). (C) Immunofluorescence images showing KCC2 and NeuN expression in the hippocampal CA1 region. Scale bars: 50 μm. (D) Quantitative analysis of membrane KCC2 labeling density to NeuN, normalized to the sham group (Sham: *N* = 4, MCAO‐R *N* = 7, MCAO‐*R* + Mino: *N* = 6). (E) I‐V plots present the experimental determination of *E*_*GABA*_ recorded with a whole‐cell patch clamp. (F) Statistical analysis identified a partially positive shift in *E*_*GABA*_ after MCAO‐R, which was reversed by minocycline‐inhibiting microglial activation. (G–I) demonstrate the outcomes of patch clamp recordings utilizing a low‐chloride electrode solution, highlighting the significant decrease in both the amplitude and frequency of mIPSCs after MCAO‐R, with only the amplitude being restored by inhibiting microglial activation (Sham: *N* = 4, *n* = 15, MCAO‐R: *N* = 3, *n* = 17, MCAO‐*R* + Mino: *N* = 4, *n* = 19). *p < 0.05, **p < 0.01, and ***p < 0.001 compared to the Sham group; ^###^ *p* < 0.001 compared to the MCAO‐R group.

During the early post‐reperfusion period, microglia were the earliest and most robust responders [17, 18]. Under our experimental conditions, the number of Iba1^+^ cells increased markedly in ischemic regions, and microglia exhibited morphological hypertrophy (Figure 4A), whereas GFAP^+^ astrocytes showed minimal activation (Figure S5A,B), indicating that microglia are the predominant glial responders in the acute phase [52]. In the sham group, Iba1 immunoreactivity was negligible, indicating quiescent microglia. Quantitative analysis confirmed a significant increase in the activated microglia in ischemic brains compared with sham (*F*_(2,14)_ = 47.71, MCAO‐R: 328.5 ± 52.6%, *N* = 7 vs. Sham: 98.9 ± 2.7%, *N* = 4, *p* < 0.01, Figure 4C). Minocycline significantly suppressed Iba1 fluorescence intensity in CA1 compared with MCAO‐R alone (*F*_(2,14)_ = 47.71, MCAO‐R: 328.5 ± 52.6%, *N* = 7 vs. MCAO‐*R* + Mino: 192.1 ± 30.6%, *N* = 6, *p* < 0.01, Figure 4A,B).

To test whether minocycline suppresses microglia‐derived BDNF, we measured hippocampal BDNF levels 23 h after MCAO‐R. As shown in the Figure S6A, BDNF protein increased after MCAO‐R and was suppressed by minocycline in this acute post‐ischemic window. Quantitative Western blots demonstrated an acute rise of hippocampal BDNF after MCAO‐R (129.3% ± 13.5% of sham, *p* < 0.01).

We further investigate whether microglial activation after MCAO‐R in mice contributed to the neuronal mKCC2 downregulation and hence the increased seizure susceptibility. Minocycline pretreatment partially rescued the KCC2 downregulation after MCAO‐R (*F*_(2,14)_ = 39.62, KCC2: NeuN ratio in MCAO‐R: 36.4% ± 8.6% of sham, *N* = 7 vs. MCAO‐*R* + Mino: 71.6% ± 16.7% of sham, *N* = 6, *p* < 0.001, Figure 4C,D). Furthermore, minocycline pretreatment also partially reversed the depolarizing shift of *E*_*GABA*_ in hippocampal neurons, making it closer to that of the sham group (*F*_(2,55)_ = 4.37, MCAO‐R: −53.1 ± 3.3 mV, *N* = 3, *n* = 15 vs. MCAO‐*R* + Mino: −55.5 ± 5.4 mV, *N* = 4, *n* = 24, *p* = 0.21; MCAO‐R + Mino vs. Sham: −57.5 ± 3.5 mV, *N* = 4, *n* = 19, *p* = 0.30, Figure 4E,F). This pattern suggests that inhibiting microglial activation partially ameliorates MCAO‐R–induced KCC2 downregulation and dysfunction, supporting recovery of KCC2‐dependent chloride extrusion toward sham levels.

When KCC2 downregulation was blocked with minocycline, the amplitude of mIPSCs was partially reversed and no significant difference was detected when compared with the sham (*F*_(2,48)_ = 4.62, MCAO‐*R*+ Mino: 18.2 ± 4.7 pA vs. MCAO‐R: 17.0 ± 3.4 pA, *N* = 3, *n* = 17, *p* = 0.56; MCAO‐*R*+ Mino vs. Sham: 20.9 ± 2.7 mV, *p* = 0.11, Figure 4G,H), whereas the frequency remained reduced (*F*_(2,48)_ = 12.84, MCAO‐*R*+ Mino: 0.6 ± 0.4 Hz, *N* = 4, *n* = 19 vs. Sham: 0.9 ± 0.4 Hz, *N* = 3, *n* = 15, *p* < 0.001, Figure 4G,I). These results indicate that microglial activation after MCAO‐R contributes to the impairment of GABA_A_R efficiency. Inhibiting microglial activation after MCAO‐R prevents the downregulation of KCC2 and partly rescues the KCC2 dysfunction, accompanied by the depolarization of *E*_*GABA*_ and an increased amplitude of mIPSCs that are consistent with reduced seizure susceptibility after MCAO‐R.

### 3.5 MCAO‐R Induces KCC2 Downregulation and Dysfunction via the BDNF/TrkB Signaling Pathway

Previous studies have indicated that activation of the BDNF/TrkB signaling pathway is closely linked to KCC2 downregulation and dysfunction in various models of neuronal injury and epilepsy [23, 53, 54]. Our current study revealed that ischemia leads to the downregulation of membrane KCC2 and that this downregulation is linked to increased seizure susceptibility. To investigate whether the BDNF/TrkB signaling pathway induces neuronal KCC2 downregulation in MCAO‐R mice, thereby enhancing seizure susceptibility after MCAO‐R, we evaluated the impact of the blockage of the BDNF/TrkB signaling pathway by K252a pretreatment on KCC2 expression levels in primary cultured hippocampal neurons after OGD/R and in CA1 hippocampal neurons of mice subjected to MCAO‐R.

**FIGURE 5:**
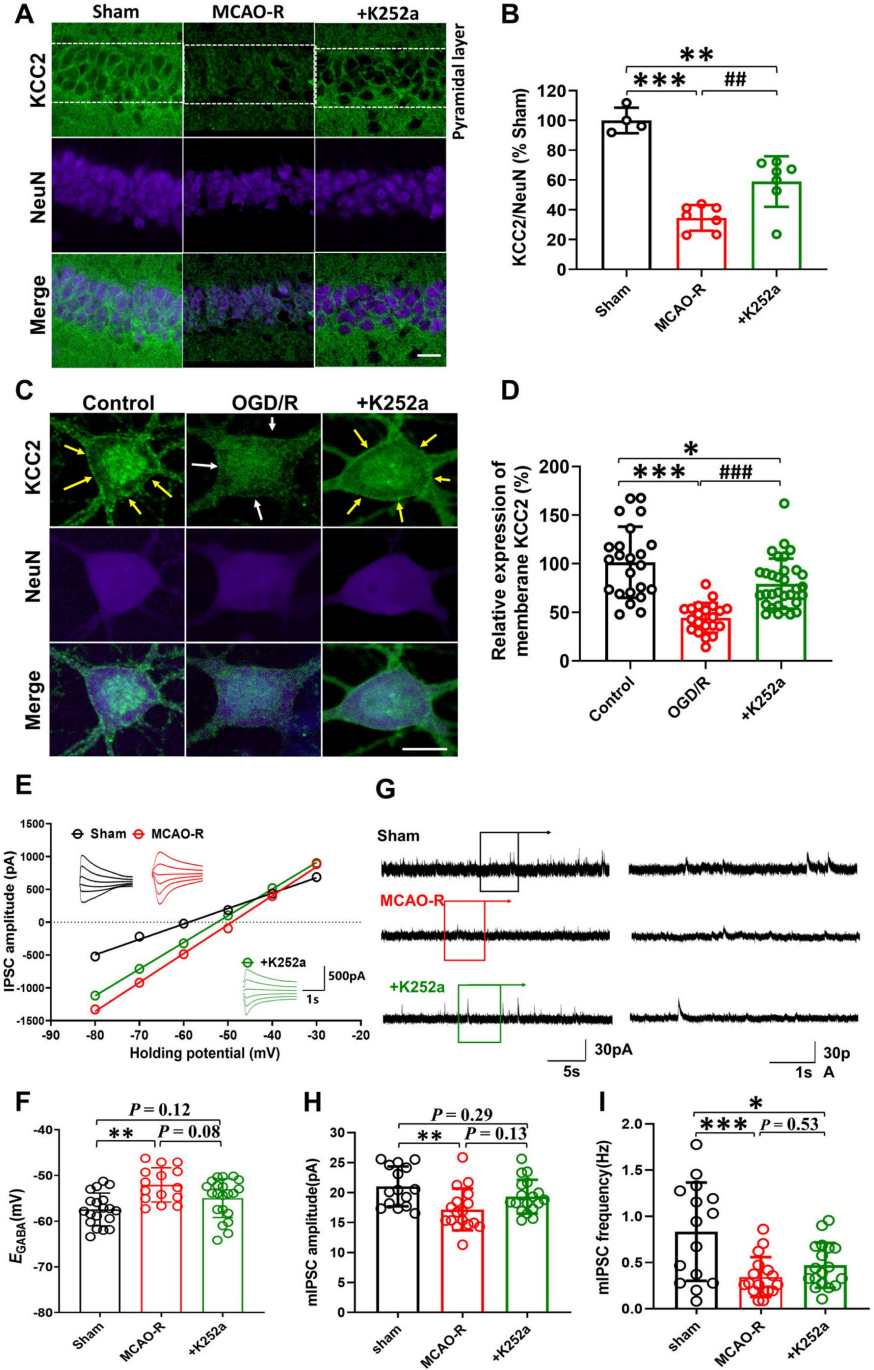
Suppression of the BDNF/TrkB signaling pathway by K252a can partly reverse the KCC2 downregulation and dysfunction after hypoxia injury. (A) NeuN and KCC2 immunolabeling in the hippocampal CA1 region of MCAO‐R mice, and those pretreated with K252a are depicted in confocal scanning images. Scale bars: 50 μm. (B) Quantitative assessment of membrane KCC2 labeling density in fluorescently labeled cells relative to NeuN (Sham: *N* = 4, MCAO‐R *N* = 7, MCAO‐*R* + K252a: *N* = 7). (C) Confocal microscopy images illustrate KCC2 (green) and NeuN (blue‐violet) expression in cultured hippocampal neurons exposed to OGD/R with or without K252a. (Control: *N* = 23 cells; OGD/R: *N* = 21 cells; OGD/*R* + K252a: *N* = 32 cells, all from *N* = 5 cultures). Scale bar: 5 μm. (D) Quantitative assessment of membrane KCC2 in cultured hippocampal neurons. (E) Experimental determination of *E*_*GABA*_ recorded by a small‐tip whole‐cell patch clamp is depicted in I‐V plots. (F) Statistical analysis indicates a significant positive shift in *E*_*GABA*_ after MCAO‐R, which is partially reversed by K252a (MCAO‐*R* + K252a: −55.0 ± 4.2 mV, *N* = 3, *n* = 21 vs. MCAO‐R: −52.1 ± 3.8 mV, *N* = 3, *n* = 15, *p* = 0.08; MCAO‐*R* + K252a vs. Sham: −57.5 ± 3.6 mV, *N* = 4, *n* = 19, *p* = 0.12). (G) Patch clamp recordings using a low‐chloride electrode solution demonstrate that both the amplitude (H) and frequency (I) of mIPSCs, which are KCC2‐dependent, are significantly reduced by MCAO‐R, with only the amplitude being partially rescued by pretreatment with K252a (MCAO‐R: 17.0 ± 3.4 pA, *N* = 3, *n* = 17 vs. MCAO‐*R* + K252a:19.3 ± 2.8 pA, *N* = 3, *n* = 18, *p* = 0.13; MCAO‐R + K252a vs. Sham:21.3 ± 3.3 pA, *N* = 3, *n* = 15). *p < 0.05, **p < 0.01, and ***p < 0.001 compared to the Sham group; ^##^ *p* < 0.01 and ^###^ *p* < 0.001 compared to the MCAO‐R group or OGD/R group.

The results showed that the downregulation in KCC2 expression after hypoxia injury was reversed when K252a blocked the BDNF/TrkB signaling pathway. In vivo, K252a pretreatment increased in KCC2 expression compared to the MCAO‐R alone (*F*_(2,15)_ = 34.20, KCC2: NeuN ratio in MCAO‐*R* + K252a: 58.9% ± 17.0% of sham, *N* = 7 vs. MCAO‐R: 34.5% ± 8.6% of sham, *N* = 7, *p* < 0.01; Figure 5A,B). Similarly, in vitro, OGD/R‐induced downregulation of mKCC2 was reversed by K252a pretreatment (*F*_(2,73)_ = 24.17, OGD/*R* + K252a: 79.4% ± 25.9%, *n* = 32; OGD/R: 44.3% ± 15.3%, *n* = 21, *p* < 0.001, Figure 5C,D). K252a pretreatment partly reversed the significant depolarizing shift in *E*_*GABA*_ that appeared in neurons after the MCAO‐R, and the difference from the sham group was not statistically significant (*F*_(2,52)_ = 8.00, MCAO‐*R* + K252a: −55.0 ± 4.2 mV, *N* = 3, *n* = 21 vs. MCAO‐R: −52.1 ± 3.8 mV, *N* = 3, *n* = 15, *p* = 0.08; MCAO‐*R* + K252a vs. Sham: −57.5 ± 3.6 mV, *N* = 4, *n* = 19, *p* = 0.12, Figure 5E,F), suggesting that blocking the BDNF/TrkB signaling pathway could partly reverse the MCAO‐R‐induced KCC2 dysfunction.

When the BDNF/TrkB signaling pathway was blocked with K252a, mIPSCs showed a trend toward increased amplitude (*F*_(2,47)_ = 5.79, MCAO‐R: 17.0 ± 3.4 pA, *N* = 3, *n* = 17 vs. MCAO‐*R* + K252a: 19.3 ± 2.8 pA, *N* = 3, *n* = 18, *p* = 0.13; MCAO‐*R* + K252a vs. Sham: 21.3 ± 3.3 pA, *N* = 3, *n* = 15, *p* = 0.29, Figure 5G,H), whereas the frequency remained reduced (*F*_(2,47)_ = 8.45, MCAO‐R: 0.3 ± 0.2 Hz, *n* = 17 vs. MCAO‐*R* + K252a: 0.5 ± 0.2 Hz, *n* = 18, *p* = 0.53; MCAO‐R + K252a vs. Sham: 0.8 ± 0.5 Hz, *n* = 15, *p* = 0.012, Figure 5G,I). These findings suggest that MCAO‐R leads to impaired mKCC2‐dependent GABA_A_R efficiency, potentially mediated through the BDNF/TrkB pathway.

### 3.6 Blocking the BDNF/TrkB Signaling Pathway and Microglial Activation Ameliorates MCAO‐R–Associated Seizure Severity

Given the role of reduced membrane KCC2 expression in seizures and epileptogenesis, and the ability of K252a to attenuate OGD/R‐induced mKCC2 downregulation in vitro, we further examined whether inhibiting the BDNF/TrkB signaling with K252a reduces OGD/R‐induced epileptiform bursts in hippocampal neurons and reduces MCAO‐R‐induced increased seizure susceptibility in mice.

**FIGURE 6:**
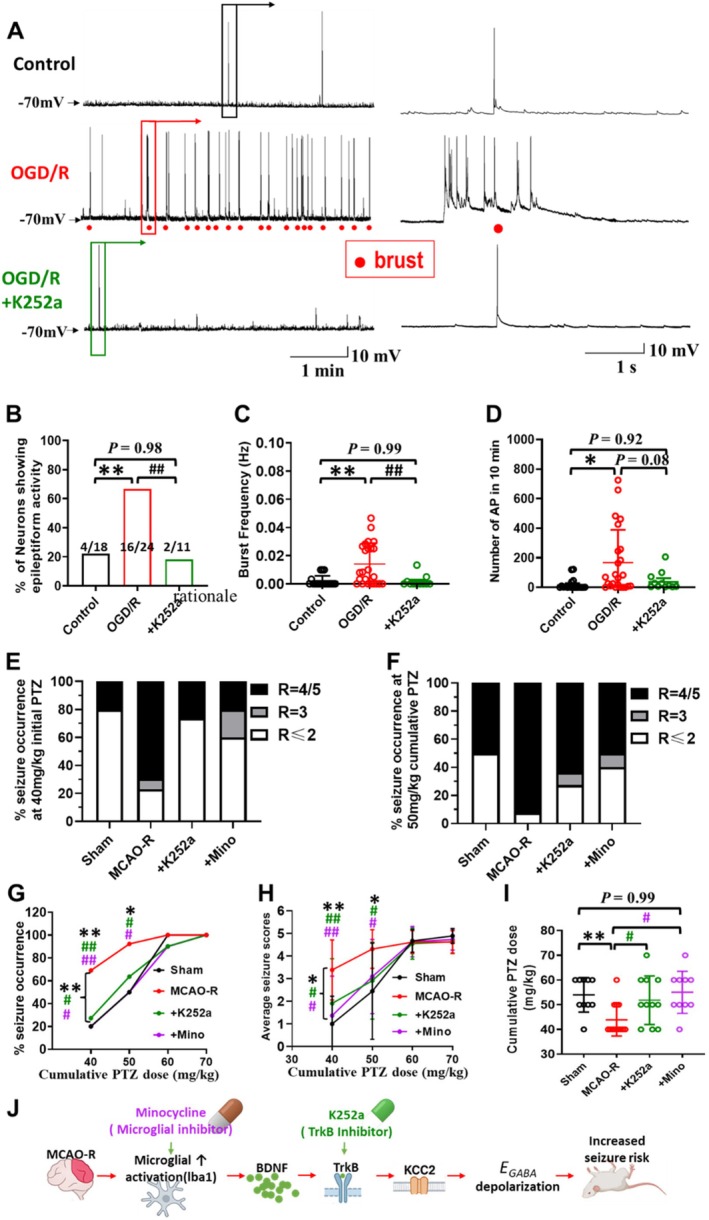
Alleviating the severity of MCAO‐R‐induced seizures by blocking the BDNF/TrkB signaling and suppressing microglial activation. (A) Representative traces demonstrate the effective suppression of epileptiform burst firing induced by OGD/R in cultured hippocampal neurons by K252a. (B–D) Bar graphs present quantitative analysis, indicating that blocking the BDNF/TrkB signaling pathway with K252a significantly reduces the percentage of neurons exhibiting epileptiform bursting firing (B), the bursting frequency (C), and the total number of APs during 10 min (D) compared to control neurons (Control: *N* = 18 cells, OGD/R: *N* = 24 cells; FUR+OGD/R: *N* = 11 cells, all from *N* = 3 cultures). (E, F) Pretreatment with K252a or minocycline significantly decreases the occurrence of Racine IV‐V seizures at 40 mg/kg (E) and 50 mg/kg (F) cumulative PTZ dose. The distribution of seizure scores among mice is represented by the R2, R3, R4, and R5. (G‐H) Line charts reveal that K252a or minocycline reduced the percentage of MCAO‐R mice with Racine IV‐V seizures (G) and the average seizure score (H) at a cumulative PTZ dose of 40 and 50 mg/kg. (I) The cumulative PTZ dose required to induce epileptic behaviors with a Racine IV‐V is increased by blocking the BDNF/TrkB signaling pathway and suppressing microglial activation (Sham: *N* = 10, MCAO‐R: *N* = 13, MCAO‐*R* + K252a: *N* = 11, MCAO‐R + Mino: *N* = 10). (J) Simplified schematic summarizing that MCAO‐R induces microglial activation (↑Iba1) and BDNF release, leading to TrkB activation, KCC2 downregulation, a depolarizing shift in *E*_*GABA*_ and increased seizure risk, whereas minocycline and K252a act upstream at microglia and TrkB, respectively, to ameliorate this cascade. *p < 0.05 and **p < 0.01 compared to the Sham group; ^#^ *p *< 0.05 and ^##^ *p* < 0.01 compared to the MCAO‐R group.

As shown in Figure 6A, the representative traces demonstrate that K252a pretreatment effectively reduced the bursting activity in primary cultured neurons after OGD/R. This inhibitory effect was demonstrated by a decrease in the percentage of bursting neurons (OGD/R: 66.7%, 16 out of 24; OGD/*R* + K252a: 18.2%, 2 out of 11, *p* < 0.01, Figure 6B), a decrease in bursting frequency (*F*_(2,50)_ = 9.04, OGD/R: 0.014 ± 0.015 Hz, *n* = 24; OGD/*R* + K252a: 0.002 ± 0.004 Hz, *n* = 11, *p* = 0.006, Figure 6C), and a decreasing trend in the total number of APs (*F*_(2,50)_ = 5.36, OGD/R: 166.8 ± 221.9, *n* = 24 vs. OGD/*R* + K252a: 42.7 ± 62.7, *n* = 11, *p* = 0.08, Figure 6D). These findings suggest that blocking the BDNF/TrkB signaling significantly inhibits OGD/R‐induced epileptiform bursts, and suggest potential implications for reducing MCAO‐R‐induced increased seizure susceptibility.

Considering that microglial activation and subsequent BDNF release are prominent after CNS injury [55, 56], and that inhibiting microglial activation prevents the downregulation of KCC2 and partially restores the functional deficits associated with KCC2 after MCAO‐R, microglial activation may play a crucial role in regulating seizure susceptibility following MCAO‐R. Our behavioral data showed that pretreatment with minocycline significantly inhibited the MCAO‐R‐induced increase in seizure susceptibility. The incidence of Racine IV‐V seizure behaviors at both the initial dose of 40 mg/kg PTZ (MCAO‐*R* + mino: 20% vs. MCAO‐R: 69%, *p* = 0.004, Figure 6E) and the cumulative 50 mg/kg PTZ (MCAO‐*R* + mino: 50% vs. MCAO‐R: 92%, *p* = 0.032, Figure 6F,G). Mean epileptic behavioral scores were also reduced in mice treated with minocycline (40 mg/kg: MCAO‐R:3.4 ± 1.4 vs. MCAO‐*R* + Mino:1.4 ± 1.7, *p* = 0.005; 50 mg/kg: MCAO‐R:4.3 ± 0.8 vs. MCAO‐R + Mino:3.1 ± 1.6, *p* = 0.029, Figure 6H). Additionally, the cumulative PTZ dose required to induce Racine IV–V seizures was higher in minocycline‐pretreated mice than in MCAO‐R alone (*t*_(21)_ = 3.57, MCAO‐*R* + Mino: 55.0 ± 8.5 mg/kg, *N* = 10 vs. MCAO‐R: 44.0 ± 6.5 mg/kg, *N* = 13, *p* = 0.002, Figure 6I), suggesting that suppressed microglial activation results in a significant increase in seizure threshold of MCAO‐R mice.

In addition, our findings indicate that blocking the BDNF/TrkB signaling pathway through K252a pretreatment partially reduced the incidence and severity of epileptic behaviors induced by PTZ in MCAO‐R mice. At both the initial dose of 40 mg/kg PTZ and the cumulative dose of 50 mg/kg PTZ, the proportion of Racine IV‐V mice with seizures in the K252a pretreatment group was lower than that in the MCAO‐R alone group (40 mg/kg PTZ: MCAO‐R: 69% vs. MCAO‐*R* + K252a: 27%, *p* = 0.041, Figure 6F; 50 mg/kg PTZ: MCAO‐R: 92% vs. MCAO‐*R* + K252a: 64%, *p* = 0.038; Figure 6G). Mean Racine scores were also lower in the K252a pretreatment group (40 mg/kg PTZ: MCAO‐R: 3.4 ± 0.7 vs. MCAO‐*R* + K252a: 2.1 ± 0.8, *p* = 0.041; 50 mg/kg PTZ: MCAO‐R: 4.3 ± 0.4 vs. MCAO‐R + K252a: 3.0 ± 0.7, *p* = 0.038; Figure 6H), suggesting that the inhibition of BDNF/TrkB signaling by K252 resulted in a reduction in the severity of PTZ‐mediated seizures in MCAO‐R mice. However, the cumulative PTZ dose at which Racine IV‐V behaviors occurred was slightly higher in the K252a pretreated group, suggesting a partial suppression of seizure susceptibility. The cumulative PTZ dose at which Racine IV‐V behaviors occurred was also higher in the K252a group (*t*_(22)_ = 2.38, MCAO‐R: 43.9 ± 6.5 mg/kg, *N* = 13 vs. MCAO‐*R* + K252a: 51.8 ± 9.8 mg/kg, N = 11, *p* = 0.03, Figure 6I), consistent with partially suppressed seizure susceptibility after MCAO‐R.

In conclusion, the results in vivo and in vitro showed that the blockade of the BDNF/TrkB signaling pathway could reverse the increased seizure susceptibility after MCAO‐R and inhibit OGD/R‐induced epileptiform bursts. Blockade of microglia activation with minocycline was significantly effective in reducing the MCAO‐R‐induced increase in seizure susceptibility, suggesting that microglia activation is greatly involved in seizures after MCAO‐R (Figure 6J).

### 3.7 CLP290 Prevents KCC2 Downregulation and Reduces Seizure Susceptibility After Ischemic Injury

**FIGURE 7:**
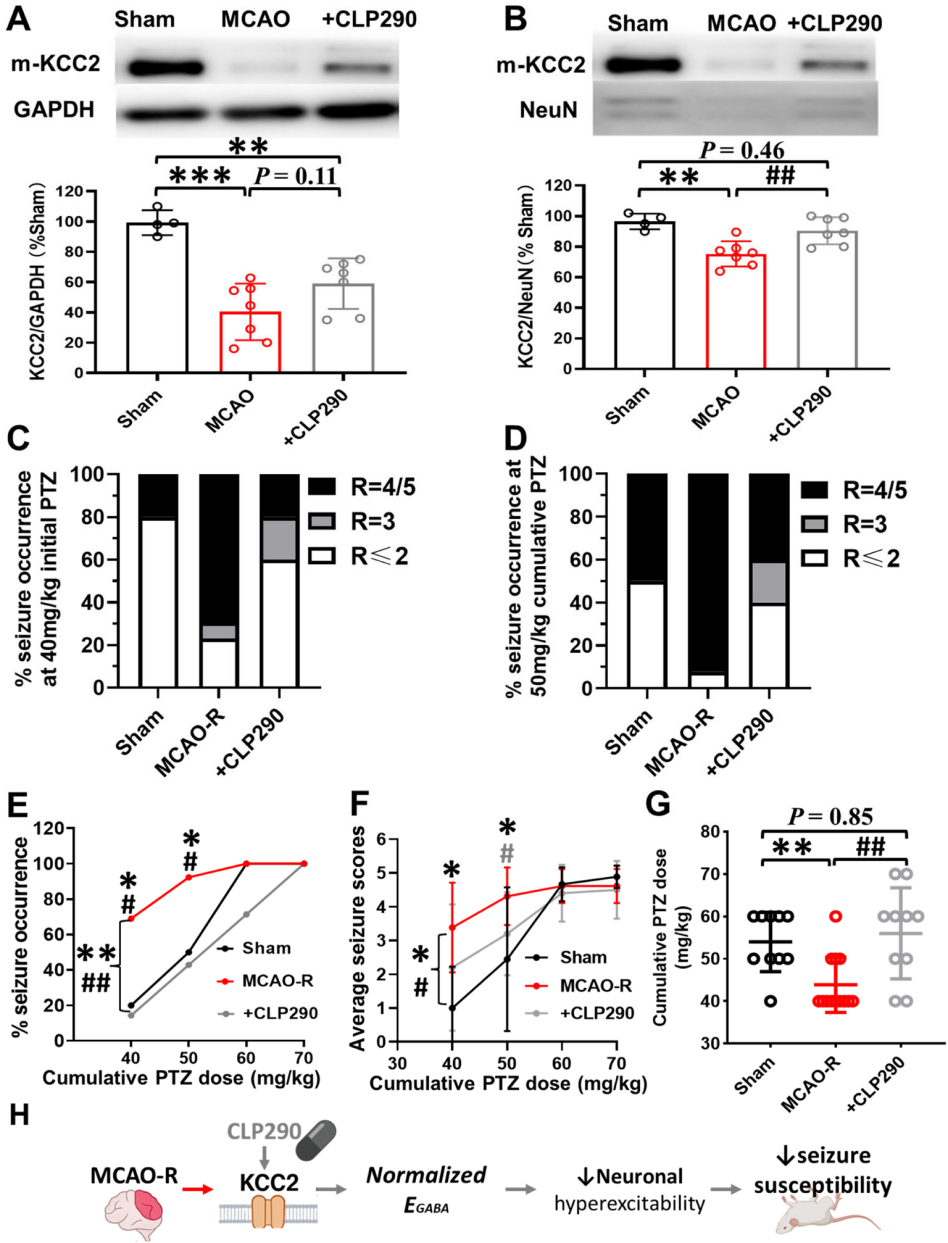
CLP290 inhibition of KCC2 expression downregulation reduces epilepsy susceptibility after MCAO‐R. (A, B) Western blot quantification of mKCC2 relative to GAPDH (A) and NeuN (B) in whole hippocampal tissue (Sham: *N* = 4, MCAO‐R: *N* = 7, MCAO‐*R* + CLP290: *N* = 7). CLP290 attenuated the downregulation of mKCC2 in surviving neurons. (C, D) Proportions of mice at each seizure level in each group at the initial PTZ dose of 40 mg/kg (C) and cumulative 50 mg/kg PTZ (D). (E) Proportion of mice with seizure Racine IV‐V at 40, 50, 60, and 70 mg/kg cumulative dose of PTZ. CLP290 pretreatment significantly reduced the proportion of MCAO‐R mice with seizure Racine IV‐V at 40 mg/kg, 50 mg/kg, and 60 mg/kg PTZ doses. (F) Mean Racine seizure scores at cumulative PTZ doses of 40, 50, 60, and 70 mg/kg. CLP290 pretreatment significantly reduced the mean seizure scores in MCAO‐R mice. (G) Cumulative PTZ dose required to reach Racine IV–V seizures in each group. CLP290 pretreatment significantly increased the cumulative dose of PTZ associated with causing seizures to Racine IV‐V (Sham: *N* = 10, MCAO‐R: *N* = 13, MCAO‐*R* + CLP290: *N* = 10). (H) Simplified schematic summarizing that CLP290, acting downstream on KCC2, restores KCC2 function after MCAO‐R, thereby normalizing *E*_*GABA*_, reducing neuronal hyperexcitability, and ultimately lowering seizure susceptibility. *p < 0.05, **p < 0.01, and ***p < 0.001 compared to the Sham group; ^#^ *p *< 0.05 and ^##^ *p* < 0.01 compared to the MCAO‐R group.

CLP290 is a small‐molecule KCC2 activator that has been reported to enhance membrane KCC2 expression and function in various models of epilepsy and neuronal injury [11, 35]. Thus, we pretreated with CLP290 before MCAO‐R surgery to verify whether CLP290 pretreatment could attenuate the KCC2 downregulation caused by ischemic injury. CLP290 significantly reduced the downregulation of mKCC2 expression in the hippocampus after MCAO‐R. When GAPDH was used as an internal reference, mKCC2 expression in MCAO‐R mice was decreased compared with sham, consistent with neuronal loss (*F*_(2,15)_ = 16.72, followed by Tukey's test, MCAO‐*R* + CLP290: 59.0% ± 16.7% of sham, *N* = 7 vs. MCAO‐R: 40.4% ± 18.7% of sham, *N* = 7, *p* = 0.11, Figure 7A). To clarify the effect of CLP290 on KCC2 expression of the surviving neurons, using NeuN as the reference. CLP290 significantly reversed the decrease in mKCC2, restoring the expression of mKCC2 to the same level as that of the Sham group (*F*_(2,15)_ = 10.78, followed by Tukey's test, MCAO‐R: 75.3% ± 8.2%, *N* = 7 vs. MCAO‐*R* + CLP290: 90.4% ± 8.9%, *N* = 7, *p* < 0.01; MCAO‐R + CLP290 vs. Sham: 96.5% ± 5.2%, *N* = 4, *p* = 0.46, Figure 7B). These data suggest that CLP290 completely inhibited the downregulation of mKCC2 expression in hippocampal surviving neurons induced by MCAO‐R.

We further explored whether inhibiting KCC2 downregulation with CLP290 reduced the seizure susceptibility in MCAO‐R mice. Behavioral data showed that CLP290 significantly suppressed the increase in seizure susceptibility induced by MCAO‐R. CLP290 pretreatment reduced the proportion of mice exhibiting Racine IV‐V seizures at both the initial 40 mg/kg PTZ dose and the cumulative 50 mg/kg PTZ doses (40 mg/kg: MCAO‐*R* + CLP290: 20% vs. MCAO‐R: 69%, *p* = 0.013; 50 mg/kg: MCAO‐*R* + CLP290: 50% vs. MCAO‐R: 92%, *p* = 0.037; 60 mg/kg: MCAO‐*R* + CLP290: 80% vs. MCAO‐R: 100%, *p* = 0.136; Figure 7C,D,E). CLP290 also reduced the mean Racine scores at 50 mg/kg cumulative PTZ dose (50 mg/kg: MCAO‐R: 4.4 ± 1.0 vs. MCAO‐*R* + CLP290: 3.2 ± 1.2, *p* = 0.028; Figure 7F). Furthermore, the cumulative dose of PTZ required for Racine IV‐V seizures was higher in the CLP290‐treated mice than in MCAO‐R alone (*F*_(2,30)_ = 7.55, MCAO‐*R* + CLP290: 56.0 ± 10.7 mg/kg, *N* = 10 vs. MCAO‐R: 44.0 ± 6.5 mg/kg, *N* = 13, *p* < 0.01; Figure 7G). CLP290 pretreatment significantly reduced the PTZ‐induced seizure severity in MCAO‐R mice (Figure 7H), indicating an increased seizure threshold with CLP290.

## 4 Discussion

**FIGURE 8:**
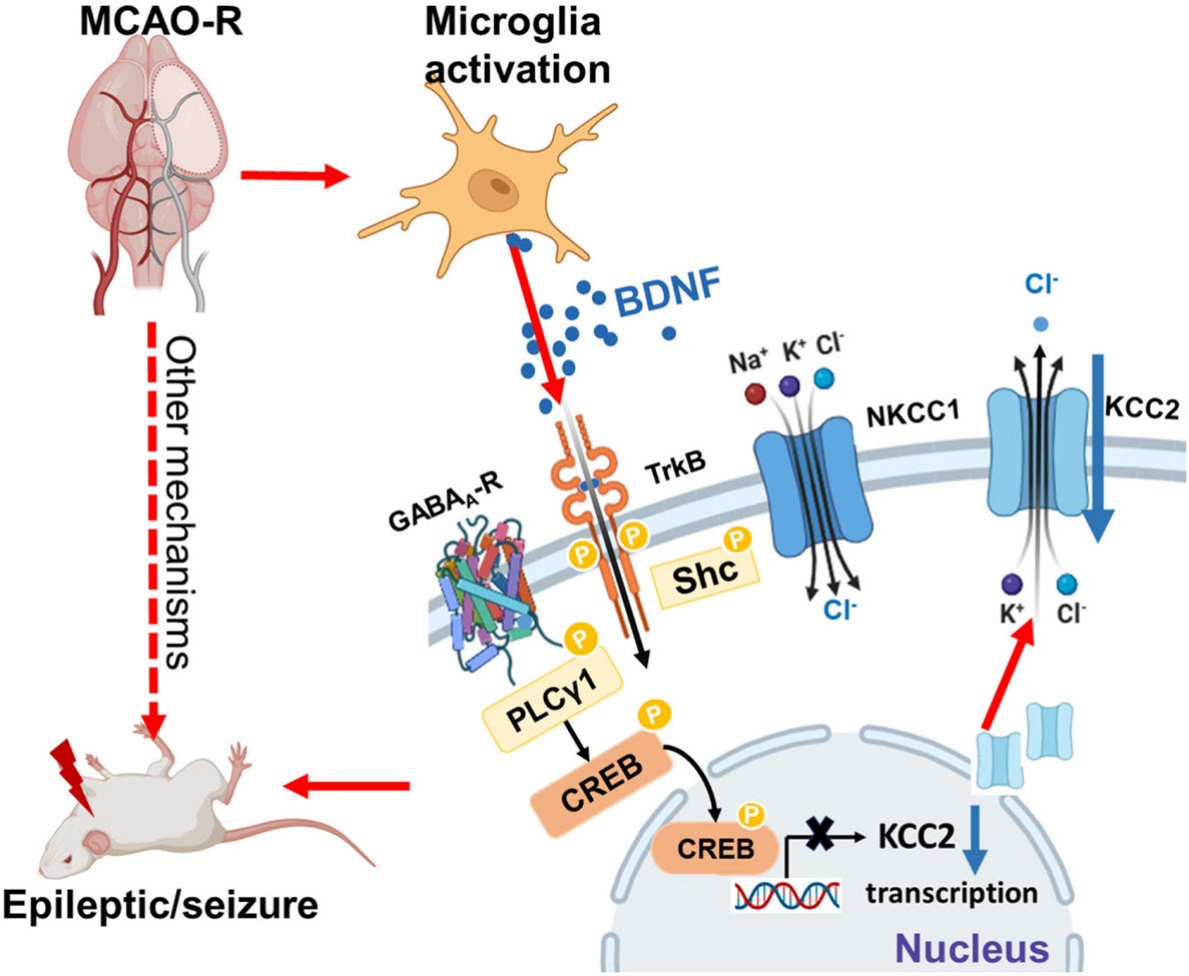
Schematic diagram of increased seizure susceptibility after MCAO‐R. Following MCAO‐R, activated microglia release BDNF, which binds to neuronal TrkB receptors and promotes TrkB autophosphorylation and downstream signaling (e.g., via CREB) [22, 23]. This signaling cascade drives transcriptional and post‐translational downregulation of the KCC2, leading to impaired chloride extrusion, increased intracellular Cl^−^ and a depolarizing shift of *E*_*GABA*_. As a result, GABA_A_R‐mediated inhibition is weakened, neuronal excitability is enhanced, and susceptibility to early post‐stroke seizures is increased. Red arrows indicate the pathological cascade from stroke to seizures, and the blue arrows emphasize the KCC2 downregulation and dysfunction as a key component of the cascade.

In this study, we provide evidence that acute ischemia–reperfusion rapidly disrupts KCC2 function in hippocampal neurons, characterized by reduced membrane KCC2 expression, a depolarizing shift in *E*_*GABA*_, diminished GABAergic inhibition, and increased neuronal excitability. Using complementary in vivo (MCAO‐R) and in vitro (OGD/R) paradigms, we further show that microglia‐derived BDNF/TrkB signaling is an important upstream driver of this chloride dysregulation, and that pharmacological interventions targeting microglial activation (minocycline), TrkB signaling (K252a), or KCC2 function itself (FUR, CLP290) can ameliorate KCC2‐dependent defects and attenuate post‐stroke seizure susceptibility. These findings support a model in which the microglia–BDNF–TrkB–KCC2 axis contributes to early post‐ischemic epileptogenesis and highlight KCC2 as a mechanism‐based therapeutic target for acute stroke‐related epilepsy, as summarized in Figure 8.

Previous work has shown that transient focal ischemia downregulates KCC2 in peri‐infarct regions, and that patients with temporal lobe epilepsy exhibit perturbed chloride gradients and depolarizing GABA responses [12, 57, 58]. Our data extend these observations by demonstrating that MCAO‐R and OGD/R produce a pronounced decrease in membrane KCC2, accompanied by a depolarizing shift of *E*_*GABA*_ and increased neuronal bursting. Western blotting revealed a marked loss of membrane‐localized KCC2 in the ipsilateral hippocampus after MCAO‐R (Figure S2), and EEG recordings showed spontaneous epileptiform discharges at baseline before PTZ administration in the MCAO‐R group (Figure S4A,B). Preventing KCC2 downregulation with FUR or CLP290 normalized *E*_*GABA*_, reduced epileptiform discharges in vitro, and increased seizure thresholds in vivo, supporting a close relationship between KCC2 function and network hyperexcitability in both hippocampal neurons and cortical networks. These results are consistent with the broader view that KCC2 is a central determinant of inhibitory efficacy and a promising anti‐seizure target [5, 9, 59]. KCC2 is a neuron‐specific chloride extruder that maintains low intracellular chloride and thereby supports hyperpolarizing GABAergic inhibition [7, 9]. Loss of KCC2 function produces an excitatory–inhibitory imbalance in hippocampal circuits and promotes epileptogenesis through chloride dysregulation [9].

Clinically, post‐stroke seizures range from early post‐stroke seizures (EPSS, often within 24 h) to late‐onset stroke‐related epilepsy that develops over weeks to months. EPSS occur in a substantial proportion of patients and are associated with an increased risk of later, often drug‐resistant epilepsy [1, 2]. In our MCAO‐R model, microglial activation, BDNF upregulation and KCC2 downregulation were most pronounced within the first 24 h, consistent with prior work showing rapid ischemia‐induced KCC2 downregulation after ischemic injury [12, 13]. These acute perturbations of chloride homeostasis are thought to initiate long‐term network instability and thus provide a rational focus on early initiator mechanisms rather than chronic maintenance phases of epilepsy.

A further contribution of this study is the spatiotemporal characterization of glial activation during the first 24 h after MCAO‐R. We observed a dense population of amoeboid Iba1^+^ microglia within the infarct core and peri‐infarct cortex, whereas GFAP^+^ astrocytes remained largely quiescent with only subtle hypertrophy at 23 h. By 72 h, both microglia and astrocytes were clearly activated, but microglial responses remained more prominent in the infarct core (Figure S5). These in vivo dynamics support the concept that microglia are the predominant early responders to ischemia, with astrocytic activation emerging later [17, 18, 19, 20]. Consistent with this timeline, hippocampal BDNF levels were significantly elevated at 23 h after reperfusion and were partially normalized by minocycline pretreatment (Figure S6A,B), indicating that microglia are a major source of the early BDNF surge. This aligns with prior reports that microglia rapidly release BDNF after CNS injury and neuropathic stimulation, thereby modulating neuronal chloride gradients and excitability [21, 60].

Downstream of microglial activation, our electrophysiological and pharmacological data implicate neuronal BDNF/TrkB signaling as a relevant regulator of KCC2 in the post‐ischemic brain. In both MCAO‐R and OGD/R paradigms, ischemia produced a consistent triad of KCC2 downregulation, a depolarizing shift in *E*_*GABA*_, and enhanced seizure susceptibility. Minocycline, administered in a regimen optimized to suppress the initial microglial response [34], reduced hippocampal BDNF levels, restored KCC2 expression, and attenuated seizures. As EEG recordings show, both K252A and minocycline treatments effectively suppressed the occurrence of Racine IV seizures following PTZ injection during the observation period (Figure S4A,B). K252a, a high‐affinity inhibitor of Trk receptor tyrosine kinases [32, 33, 41], produced similar functional rescue when applied in vivo and in vitro, consistent with reports that K252a suppresses p‐TrkB and downstream signaling [61, 62]. Although technical limitations prevented robust p‐TrkB and p‐CREB quantification in our hippocampal samples, the combination of BDNF biochemistry, chloride electrophysiology, and TrkB‐targeted pharmacology supports a framework in which microglia‐derived BDNF activates neuronal TrkB to downregulate KCC2 and depolarize *E*_*GABA*_. This is in agreement with prior work showing that excessive BDNF/TrkB signaling suppresses KCC2 and converts GABAergic transmission from inhibitory to excitatory in mature neurons [23, 31, 63].

The four pharmacological tools used in this study act at distinct levels of the microglia–BDNF–TrkB–KCC2 axis and thereby provide insight into causal relationships and pharmacological specificity. Minocycline is an upstream intervention that reduces microglial activation and BDNF release, but also exerts broader anti‐inflammatory and antioxidant actions [60, 64, 65]; it is therefore best interpreted as an upstream modulator rather than a microglia‐specific tool. K252a targets the intracellular kinase domain of TrkB, preventing autophosphorylation at Tyr706/707 and suppressing downstream ERK–CREB, PI3K–AKT, and PLCγ–CaMK pathways known to influence KCC2 expression [66, 67]. Furosemide, at the low doses used here, was employed as a probe of KCC2‐dependent inhibition. Based on prior work, such doses preferentially stabilize KCC2 function with limited NKCC1 inhibition [12, 31, 40], but some contribution of NKCC1 blockade cannot be fully excluded. We therefore interpret FUR as acting on the KCC2/NKCC1 axis rather than as a perfectly selective KCC2 agent and place greater mechanistic weight on CLP290.

CLP290 is a downstream KCC2 potentiator with strong evidence for selectivity: CLP257/CLP290 enhances KCC2 via Ser940‐dependent membrane stabilization without detectable activity on NKCC1 or other CCC transporters [35, 36]. In our model, CLP290 restored membrane KCC2 in surviving hippocampal neurons to near‐sham levels, normalized *E*_*GABA*_ and markedly reduced post‐ischemic seizure susceptibility. EEG analysis supported a disease‐modifying potential: untreated MCAO‐R mice showed synchronized Racine IV activity with high‐frequency spikes at relatively low PTZ doses, whereas CLP290‐pretreated mice maintained lower Racine stages with slower waves at substantially higher PTZ doses (Figure 7 and Figure S3). The more complete normalization achieved with CLP290, compared with the partial effects of minocycline and K252a on *E*_*GABA*_ and mIPSC amplitude, is compatible with the notion that KCC2 may serve as a downstream convergence point for BDNF/TrkB‐dependent and other neuroinflammatory pathways mediated by microglia. Neuroinflammatory cytokines such as TNF‐α and IL‐1β have been implicated in activity‐ and phosphorylation‐dependent regulation of KCC2 trafficking and stability in synaptic scaling and pain models [63, 68, 69], suggesting that multiple inflammatory signals may converge on the KCC2 axis after ischemia.

Our findings also have implications for pharmaco‐resistance in post‐stroke seizures. Roughly one‐third of patients with stroke‐related epilepsy develop drug‐resistant seizures despite conventional anti‐seizure medications [1]. When KCC2 is downregulated and GABA_A_R becomes less inhibitory or even depolarizing, further GABA potentiation may be ineffective or paradoxical, whereas restoring KCC2 function directly corrects the chloride gradient and reinstates inhibition. KCC2 activators such as CLP290 and related compounds enhance KCC2 membrane stability without systemic diuretic effects and show favorable preclinical safety [5, 35], making them attractive candidates for targeting chloride dysregulation. Furosemide can serve as a tool compound on the KCC2/NKCC1 axis [31], but its strong renal actions limit long‐term use. By contrast, strategies that dampen BDNF/TrkB signaling require caution. The BDNF/TrkB pathway supports synaptic plasticity, neuroprotection and post‐stroke recovery [70, 71], so chronic or non‐selective TrkB blockade may impair rehabilitation. Our data instead support a time‐restricted “hit‐and‐run” approach, in which TrkB signaling is transiently attenuated in the early (0–24 h) post‐ischemic window to prevent microglia‐derived BDNF–induced KCC2 downregulation while preserving later neurotrophic functions. More selective modulators that preferentially disrupt pathological BDNF–TrkB–KCC2 coupling, rather than global TrkB inhibition, will likely be needed. Likewise, although minocycline has good CNS penetration and robust anti‐microglial effects, its broad antibiotic use raises concerns about microbiome disturbance, resistance and rare immune‐mediated events [72], suggesting that it is best regarded as a proof‐of‐concept tool rather than a long‐term anti‐epileptic therapy.

Several limitations should be acknowledged. First, our study focuses on the acute/subacute phase (< 24 h), whereas stroke‐related epilepsy usually develops over weeks to months. We did not assess long‐term KCC2 dynamics, chronic glial remodeling, or spontaneous recurrent seizures. Longitudinal experiments combining chronic EEG monitoring, cell‐type‐specific manipulations, and delayed time points (e.g., 7–14 days or longer) will be required to determine whether early KCC2 rescue durably alters epileptogenesis. Second, mechanistic inferences regarding TrkB activation are based on BDNF biochemistry, electrophysiological readouts, and K252a pharmacology rather than direct quantification of p‐TrkB. Future work using more sensitive phospho‐specific assays and in situ biosensors will help validate the activation state of TrkB‐CREB pathways in defined neuronal populations. Third, our pharmacological tools are not fully specific: minocycline has pleiotropic anti‐inflammatory and antioxidant actions; we therefore interpret them as upstream modulators rather than perfectly selective probes. Microglia‐depletion strategies such as PLX3397 offer a more specific approach [73, 74] and will be important to confirm the contribution of microglia to KCC2 dysfunction. Fourth, the human relevance of KCC2 dysregulation in post‐stroke epilepsy remains incompletely defined, and direct histopathological data from peri‐infarct tissue are scarce. Although indirect evidence from human temporal lobe epilepsy (KCC2 downregulation with depolarizing GABA responses) and from peri‐infarct microglial activation with elevated BDNF in stroke and neuropathic pain [57, 58, 60] supports the plausibility of our findings and highlights an important unmet need for future translational studies.

In summary, this study provides convergent evidence that microglia‐derived BDNF/TrkB signaling is a key upstream driver of KCC2 hypofunction, chloride dysregulation, and enhanced seizure susceptibility in the acute phase after ischemic stroke. Targeting this axis at multiple levels, including microglial activation, TrkB kinase activity, and KCC2 function, restores inhibitory control and attenuates post‐stroke seizures. These findings refine our understanding of glia–neuron crosstalk in early epileptogenesis and highlight the microglia–BDNF–TrkB–KCC2 pathway as a promising therapeutic target for stroke‐related epilepsy.

## Funding

National Natural Science Foundation of China (32111530119, 31771188, 81971204); Shanghai Municipal Science and Technology Major project (No. 2018SHZDZX01) and ZJLab, Sanming Project of Medicine in Shenzhen (No. SZSM202111010) and Shenzhen Key Medical Discipline Construction Fund (No. SZXK048). This study was partly supported by a grant from the Shenzhen Science and Technology Innovation Commission Municipality (JCYJ20210324103409023) to LW. KC and BHW also received an award from Xiyuan Project of the Fudan University Undergraduate Research Opportunities Program (FDUROP, 2021–2022).

## Ethics Statement

All animal procedures were performed in accordance with institutional guidelines for animal care and use, adhered to the ARRIVE recommendations, and were approved by the following institutional ethics committees: Fudan University Animal Ethics Committee (Approval ID: No. 20170223‐102). Shenzhen Second People's Hospital Animal Ethics Committee (Approval ID: No. 202200106).

## Conflicts of Interest

The authors declare no conflicts of interest.

## Supporting information


**Data S1:** Supporting Information.

## Data Availability

Data will be made available on request.
