# Supplementary material for: KCC2 Dysfunction Mediated by Microglial BDNF/TrkB Signaling Exacerbates Early Post‐Stroke Seizure Susceptibility
Source: CNS Neurosci Ther. 2026 Feb 13;32(2):e70795. doi: 10.1002/cns.70795 (PMC12905008; doi:10.1002/cns.70795)
Supplement: Supplementary file 1 — Data S1: Supporting Information. [file CNS-32-e70795-s001.docx]

# KCC2 dysfunction mediated by microglial BDNF/TrkB signaling exacerbates early post-stroke seizure susceptibility

Jing Zhou^1,2,#^, Benjamin H Wang^1,#^, Jiangning Yu^1,#^, Guoxiang Wang^1,#^, Jingyi Cai^1^, Mohan Yu^1,$^, Kehua Chen^1^, Li Wan^2,*^, Xu Liu^1,*^, Zhigang Yang ^1,*^, Yulong Wang^2,*^, Yun Wang^1,*^

^1^ Department of Neurosurgery and Neurology, Institutes of Brain Science, State Key Laboratory of Medical Neurobiology and MOE Frontiers Center for Brain Science, Institute of Biological Science, Zhongshan Hospital, Fudan University, NO. 131 Dongan Road, Xuhui District, Shanghai 200032, China.

^2^ Department of Rehabilitation, the First Affiliated Hospital of Shenzhen University / Shenzhen Second People’s Hospital, Guangdong Province, NO. 3002 Sungang West Road, Futian District, Shenzhen 518035, China.

^#^ Equal contribution.

^$^ Current address: Concord College, Acton Burnell, Shropshire, Shrewsbury SY5 7PF， UK

Supplementary File


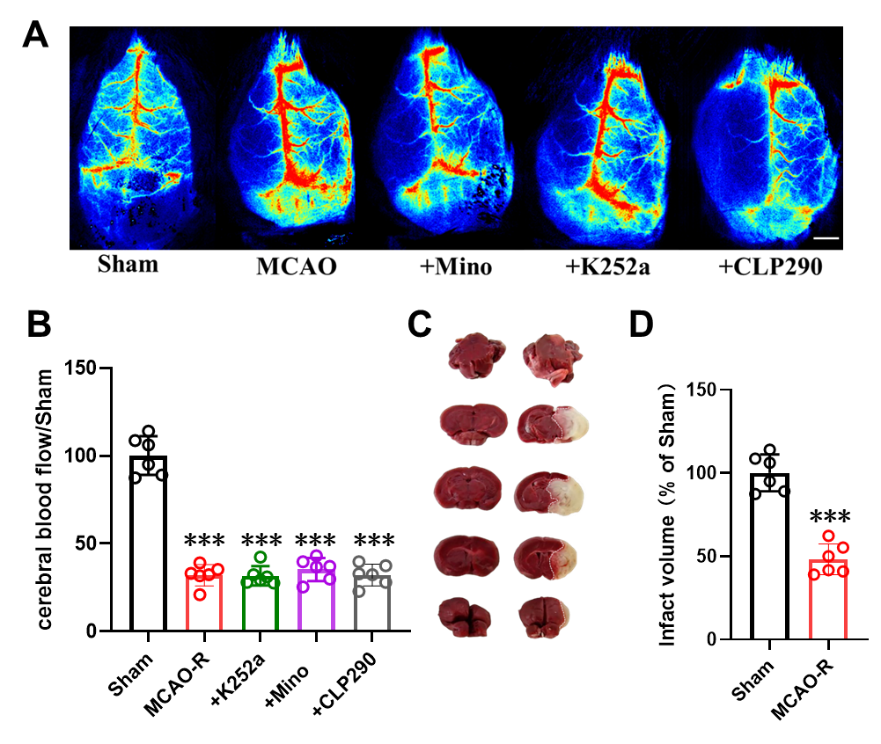


**Figure S1**: **Representative images of cerebral blood flow and infarct volume in MCAO-R mice.** (A) Laser speckle contrast imaging (LSCI) maps of cerebral blood flow in the cortex of mice subjected to different treatments: Sham, MCAO-R, MCAO-R + FUR, MCAO-R + CLP290, and MCAO-R + Minocycline. The color scale (blue to red) represents relative CBF, with red indicating high perfusion and blue indicating low perfusion. The white dashed line delineates the ischemic core. (B) Representative TTC-stained brain sections from the same groups, showing the extent of infarction (pale area) 24 hours post-surgery. (N = 5–6 per group). ****P* < 0.001 vs. Sham.


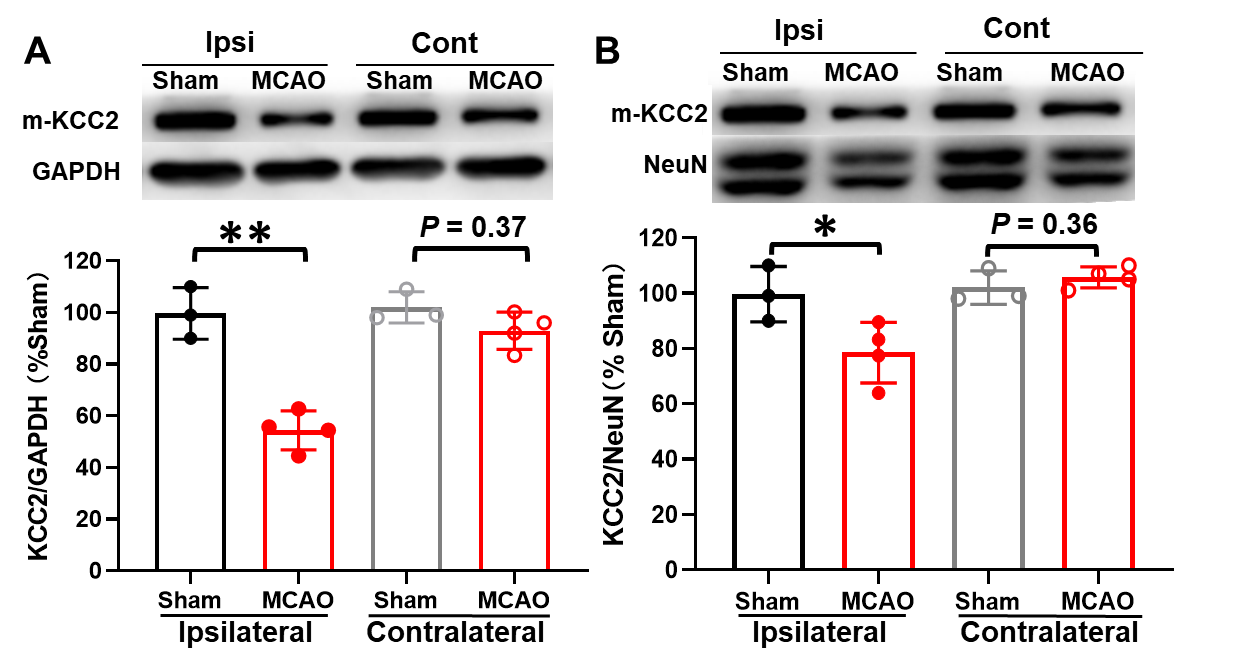


**Figure S2:** Western blot images of membrane KCC2, GAPDH, and NeuN in hippocampal homogenates from sham and MCAO mice. Ipsilateral refers to ischemic injury on the same side, while contralateral refers to ischemic injury on the opposite side.


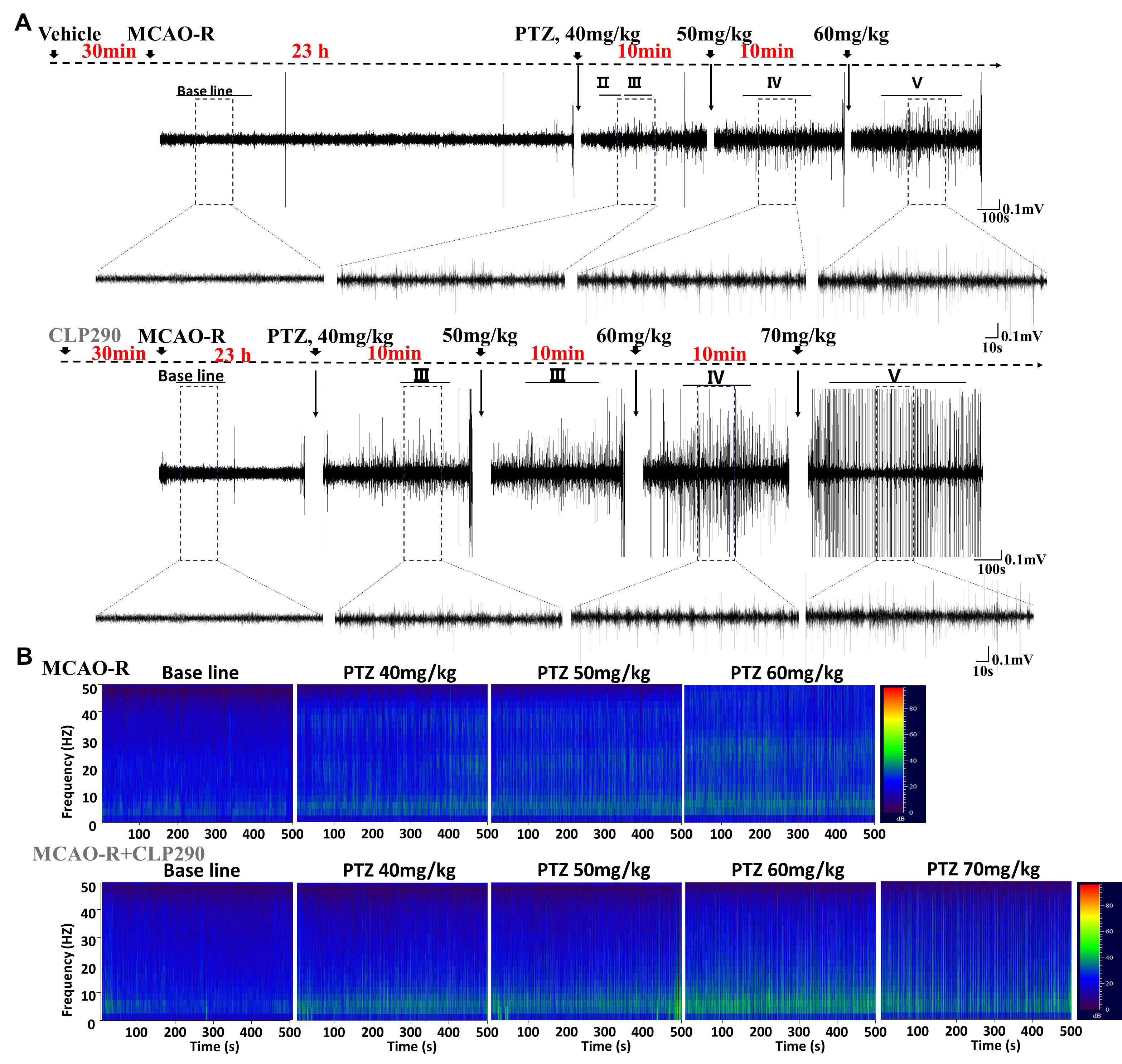


**Figure S3:** (A) Representative EEG recordings demonstrate improvements in PTZ-induced acute seizures with CLP290 intervention. The lower traces are enlarged views of the dashed boxes in the upper traces, with the black dashed box indicating intermittent Racine scores. (B) The power spectrum revealed that high-frequency firing intensity decreased over 40 minutes and did not attain Racine levels IV-V after initial PTZ injection in the CLP290 pretreatment group, in contrast to the untreated MCAO-R group. **P* < 0.05, **P < 0.01, and ***P < 0.001 compared to the Sham group; ###P < 0.001 compared to the MCAO-R group.


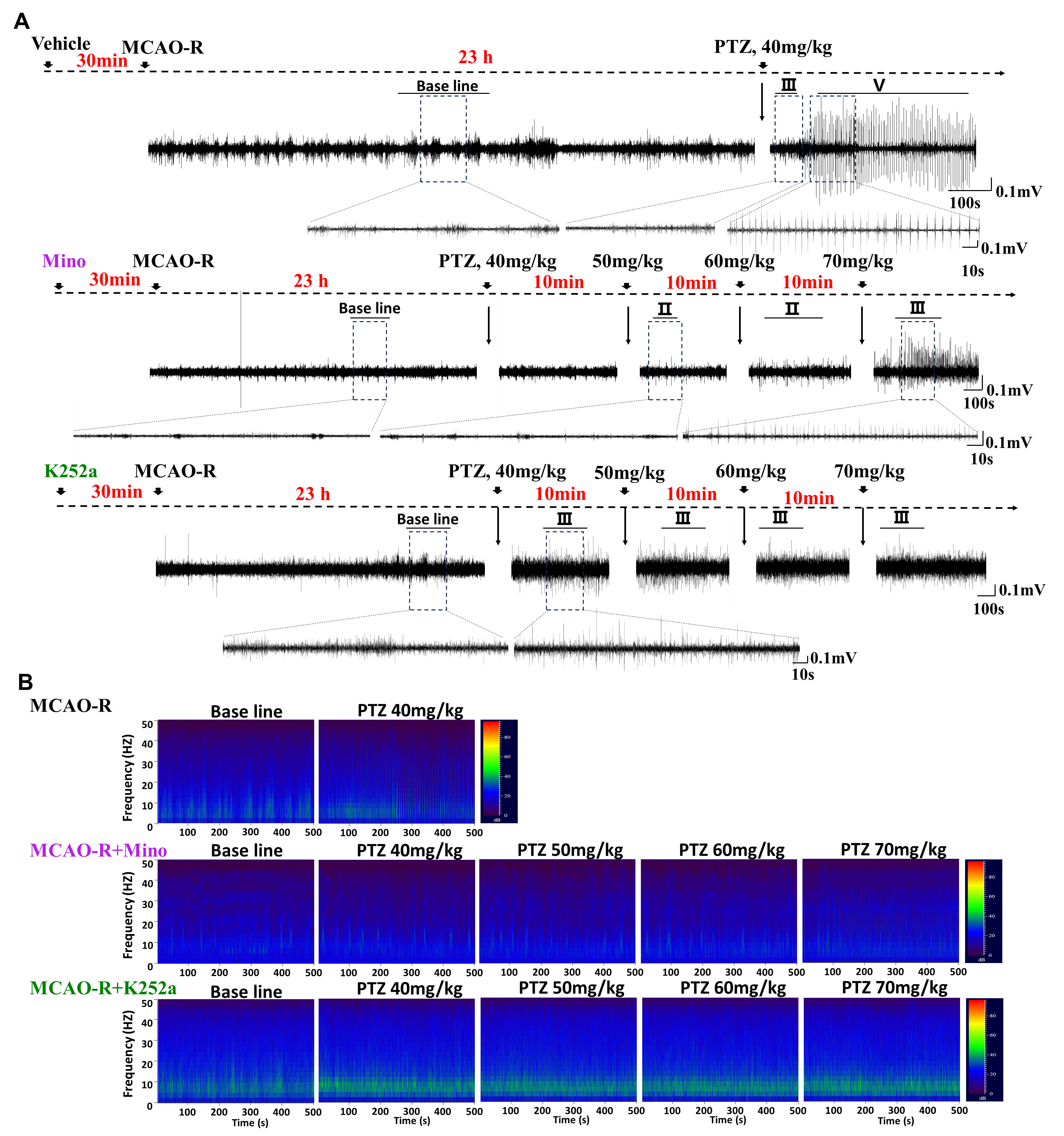


**Figure S4:** (A) Representative EEG recording traces from MCAO-R mice pre-treated with K252a or minocycline. The lower traces provide an enlarged view of the dashed boxes in the upper traces. (B) EEG power derived from these recordings was plotted against time, ranging from PTZ administration to the subsequent 500 seconds. In MCAO-R mice pre-treated with K252a or minocycline, the intensity of high-frequency firing exhibited a decline during the initial 10 minutes and did not reach Racine levels IV-V during the 40-minute analysis following initial PTZ injection, in comparison to untreated MCAO-R group. Statistical significance is denoted as follows: *P < 0.05, **P < 0.01, and ***P < 0.001 compared to the Sham group; ^###^P < 0.001 compared to the MCAO-R group.


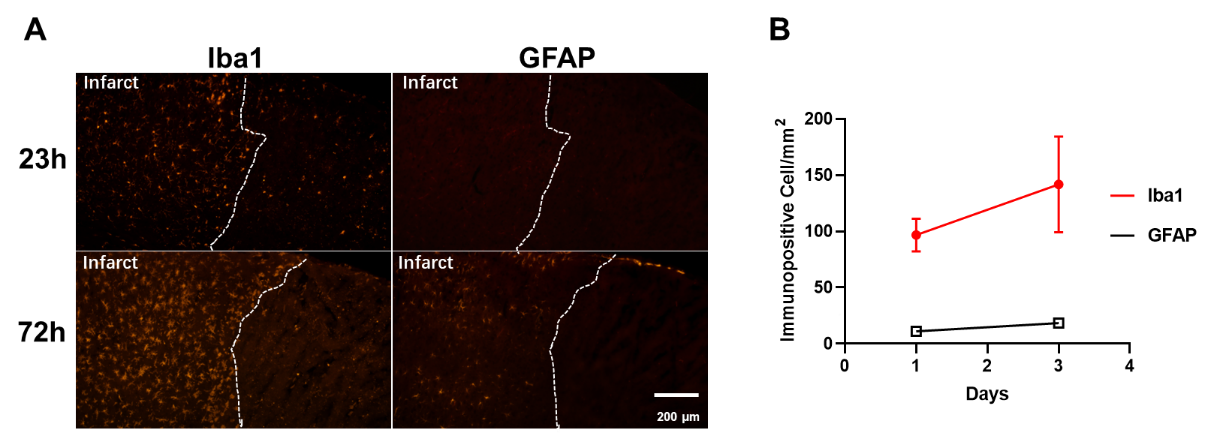


**Figure S5: Spatiotemporal dynamics of microglial and astrocytic activation in the MCAO-R model.** (A) Representative immunofluorescence images of the microglial marker Iba1 (left) and the astrocytic marker GFAP (right) in the peri-infarct cortex at 23 h and 72 h after MCAO-R. At 23 h, numerous activated, amoeboid Iba1⁺ microglia are already present within and around the infarct core, whereas GFAP⁺ astrocytes show only mild changes with limited hypertrophy. By 72 h, microglial activation remains robust and astrocytes also display clear reactive changes. (B) Quantification of Iba1⁺ cell density within the infarct core at 23 h and 72 h (N = 3 per time point), confirming a marked increase in microglial activation over time. Taken together, these data indicate that microglia are the predominant early responders to ischemic injury, with astrocytic activation emerging later. The white dashed line delineates the border between the infarct core (left) and peri-infarct penumbra (right). Scale bar = 200 μm.


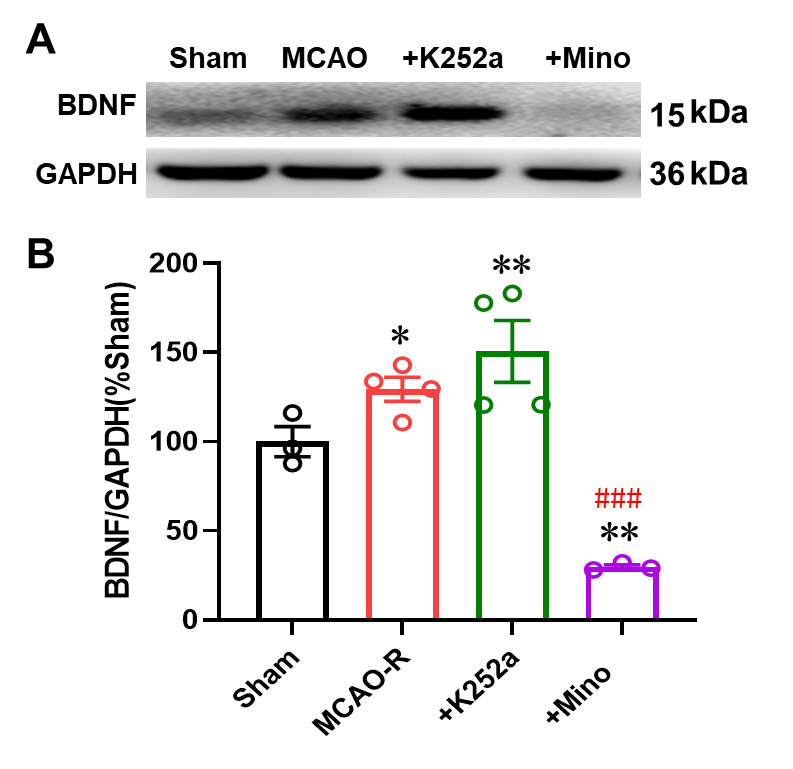


**Figure S6: Minocycline attenuates the early increase in hippocampal BDNF after MCAO-R.** (A) Representative Western blots for BDNF in hippocampal lysates 23 h after reperfusion, with GAPDH as a loading control. (B) Quantification of BDNF/GAPDH ratios normalized to Sham. Data are presented as mean ± SD (N = 3–4 mice per group). **P* < 0.05, ***P* < 0.01 vs. Sham; ##*P* < 0.01, ###*P* < 0.001 vs. MCAO-R (one-way ANOVA followed by Tukey’s post hoc test).

Supplementary Methods

Electrophysiology recordings

Experiments involving whole-cell patch-clamp recordings were performed on hippocampal CA1 pyramidal neurons using an Axon 700B amplifier and a Digidata 1440A converter. Data were acquired using pCLAMP 10.2 software. Cell morphology in the CA1 region of the hippocampus was initially examined with a 10× objective on an upright Nikon microscope. Because of ischemia-induced cell death in some regions of MCAO-R mice, the field of view was continuously adjusted to locate surviving cells around the affected area before recording. Surviving cells in the CA1 region were then examined using a 40× water-immersion objective. Micromanipulation under visual guidance on the monitor was used to identify and select brightly refractile, morphologically intact, cone-shaped pyramidal neurons for recording.

Series resistance (R_s_) was continuously monitored throughout the recordings using brief hyperpolarizing test pulses (5–10 mV, 10 ms) delivered at regular intervals. Recordings were accepted only if the initial R_s_ was ≤ 20–25 MΩ and did not vary by more than 20% during the experiment; cells that failed to meet these criteria were excluded from analysis. R_s_ was partially compensated (60–80%) using the amplifier circuitry, and data showing obvious loss of voltage control were discarded. The liquid junction potential between the internal solutions and ACSF was estimated using the pCLAMP junction potential calculator to be ~10 mV (pipette negative) and was not corrected; all membrane potentials, including *E_GABA_*, are therefore reported without junction potential correction.

Patch electrodes had the following specifications: outer diameter 1.50 mm, inner diameter 0.86 mm, length 10 cm, and were pulled with an M-285 horizontal puller (Sutter) before use. Optimal impedance (2–6 MΩ) was achieved after filling the electrode with internal solution. A slight positive pressure was applied before electrode insertion into the bath solution to prevent tip blockage. Gentle advancement of the micromanipulator and gradual release of positive pressure near the target cell allowed formation of a high-resistance (> 1 GΩ) gigaseal with the cell membrane. Whole-cell recordings were initiated after a stable seal was obtained, with a holding potential of −70 mV, and membrane rupture was achieved by brief suction.

The perforated patch-clamp technique, which is the conventional method for recording *E_GABA_*_,_ is time-consuming and relatively inefficient. Therefore, based on previous experience and published work, we used a Cl⁻-permeable, high-impedance (10–15 MΩ) whole-cell patch-clamp configuration to record *E_GABA_* in a comparable manner ^1,2^. The internal solution contained (in mM): 125 K-gluconate, 10 KCl, 0.5 EGTA, 10 HEPES, 2 Mg-ATP, 0.5 Na-GTP, and 10 Na₂-phosphocreatine, adjusted to pH 7.3 with an osmolarity of ~300 mOsm. DNQX (20 μM; Abcam, USA), NBQX (1 μM; Abcam, USA), D-AP5 (50 μM; Abcam, USA), and TTX (1 μM; Hebei Academy of Fishery Sciences, China) were added to the ACSF to block glutamate receptors and Na⁺ channels during recording. After identifying surviving cells in the pyramidal cell layer of the CA1 region under a 10× objective, a puffer pipette for GABA application was positioned ~10 μm above and ~20 μm lateral to the target neuron. After membrane rupture and establishment of whole-cell access, the membrane potential of the neuron was stepped from −80 mV to −30 mV in 10-mV increments every 2 s. In the vicinity of the recording electrode on the CA1 pyramidal neuron, GABA (250 μM, 50 ms, 50 psi; Sigma, USA) was pressure-ejected, and the resulting GABA_A_R-mediated currents were recorded. The I–V relationship was constructed and fitted by linear regression to determine the membrane potential at which the current was 0 pA, corresponding to the reversal potential of GABA_A_R-mediated currents (*E_GABA_*).

For recording miniature inhibitory postsynaptic currents (mIPSCs), patch pipettes (3–5 MΩ) were used as previously described ^3^. Neurons were voltage-clamped at 0 mV. Patch pipettes were filled with a cesium-based internal solution containing (in mM): 127.5 CsMeCO₃, 7.5 CsCl, 10 HEPES, 0.6 EGTA, 2.5 MgCl₂·6H₂O, 4 Na₂ATP, 0.4 Na₃GTP, 10 Na₂-phosphocreatine, and 5 QX-314, adjusted to pH 7.3 with KOH (~300 mOsm). To isolate GABAA receptor–mediated mIPSCs, DNQX (20 μM), D-AP5 (50 μM), and TTX (1 μM) were added to the ACSF to block AMPA/kainate receptors, NMDA receptors, and voltage-gated Na⁺ channels, respectively.

In experiments involving cultured hippocampal neurons, FUR (100 μM) and K252a (100 nM) were applied before the OGD procedure. For recordings, coverslips were transferred to a recording chamber containing extracellular solution with the following composition (in mM): 128 NaCl, 5 KCl, 30 D-glucose, 25 HEPES, 2 CaCl₂, and 1 MgCl₂, pH 7.3 (~300 mOsm). Patch pipettes (3–5 MΩ) were filled with an internal solution containing (in mM): 125 potassium gluconate, 10 KCl, 5 EGTA, 10 HEPES, 10 phosphocreatine, 4 Mg-ATP, and 0.5 Na-GTP, pH 7.3 (~300 mOsm). To elicit seizure-like activity, whole-cell current-clamp recordings were performed as previously described ^4,5^. The membrane potential of neurons was maintained at approximately −70 mV during current-clamp recording. Epileptiform activity was defined as a depolarizing shift exceeding 10 mV in amplitude and lasting at least 300 ms, with a minimum of five action potentials riding on the depolarizing envelope ^4^. Neurons were classified as “bursting” if they exhibited repetitive burst activity at least twice within a 10-minute recording interval ^4,6^.

Reference

1. Xu C, Zhao MX, Poo MM, Zhang XH. GABA(B) receptor activation mediates frequency-dependent plasticity of developing GABAergic synapses. *Nature neuroscience.* 2008;11(12):1410-1418.

2. Hewitt SA, Wamsteeker JI, Kurz EU, Bains JS. Altered chloride homeostasis removes synaptic inhibitory constraint of the stress axis. *Nature neuroscience.* 2009;12(4):438-443.

3. Yu J, Wang G, Chen Z, et al. Deficit of PKHD1L1 in the dentate gyrus increases seizure susceptibility in mice. *Hum Mol Genet.* 2023;32(3):506-519.

4. Qi J, Wang Y, Jiang M, Warren P, Chen G. Cyclothiazide induces robust epileptiform activity in rat hippocampal neurons both in vitro and in vivo. *The Journal of physiology.* 2006;571(Pt 3):605-618.

5. Liu X, Chen B, Chen L, et al. U-shape suppressive effect of phenol red on the epileptiform burst activity via activation of estrogen receptors in primary hippocampal culture. *PloS one.* 2013;8(4):e60189.

6. Wang Y, Qi JS, Kong S, et al. BDNF-TrkB signaling pathway mediates the induction of epileptiform activity induced by a convulsant drug cyclothiazide. *Neuropharmacology.* 2009;57(1):49-59.
